# Multi-Omics Analyses Characterize the Gut Microbiome and Metabolome Signatures of Soldiers Under Sustained Military Training

**DOI:** 10.3389/fmicb.2022.827071

**Published:** 2022-03-25

**Authors:** Yifan Shi, Peng Wang, Da Zhou, Longchang Huang, Li Zhang, Xuejin Gao, Gulisudumu Maitiabula, Siwen Wang, Xinying Wang

**Affiliations:** ^1^Department of General Surgery, Affiliated Jinling Hospital, Medical School of Nanjing University, Nanjing, China; ^2^Department of Gastrointestinal Surgery, Affiliated Hospital of Jiangnan University, Wuxi, China; ^3^Department of General Surgery, Jinling Hospital, The First School of Clinical Medicine, Southern Medical University, Nanjing, China

**Keywords:** soldier, gut microbiome, 16S rRNA, gut metabolome, metabolism, physical performance

## Abstract

Exercise can directly alter the gut microbiome at the compositional and functional metabolic levels, which in turn may beneficially influence physical performance. However, data how the gut microbiome and fecal metabolome change, and how they interact in soldiers who commonly undergo sustained military training are limited. To address this issue, we first performed 16S rRNA sequencing to assess the gut microbial community patterns in a cohort of 80 soldiers separated into elite soldiers (ES, *n* = 40) and non-elite soldiers (N-ES, *n* = 40). We observed that the α-diversities of the ES group were higher than those of the N-ES group. As for both taxonomical structure and phenotypic compositions, elite soldiers were mainly characterized by an increased abundance of bacteria producing short-chain fatty acids (SCFAs), including *Ruminococcaceae_UCG-005*, *Prevotella_9*, and *Veillonella*, as well as a higher proportion of oxidative stress tolerant microbiota. The taxonomical signatures of the gut microbiome were significantly correlated with soldier performance. To further investigate the metabolic activities of the gut microbiome, using an untargeted metabolomic method, we found that the ES and N-ES groups displayed significantly different metabolic profiles and differential metabolites were primarily involved in the metabolic network of carbohydrates, energy, and amino acids, which might contribute to an enhanced exercise phenotype. Furthermore, these differences in metabolites were strongly correlated with the altered abundance of specific microbes. Finally, by integrating multi-omics data, we identified a shortlist of bacteria-metabolites associated with physical performance, following which a random forest classifier was established based on the combinatorial biomarkers capable of distinguishing between elite and non-elite soldiers with high accuracy. Our findings suggest possible future modalities for improving physical performance through targeting specific bacteria associated with more energetically efficient metabolic patterns.

## Introduction

The human intestine harbors a complex and diverse ecosystem comprising myriads of bacterial taxa and various viruses, archaea, fungi, and protozoa, leading to a biomass of more than 1.5 kg (Qin et al., 2010; Tierney et al., 2019). In recent decades, the gut microbiome has emerged as the focus of converging interest in human health research, primarily due to its tremendous impact on host physiology, nutrition, metabolism, and immune system development (Feng et al., 2018; Schluter et al., 2020). Accumulating evidence indicates that many factors, such as diet (Fujisaka et al., 2018; Garcia-Mantrana et al., 2018), age (Ayeni et al., 2018; Del Chierico et al., 2018; Nagpal et al., 2018), genetics (Zhou et al., 2014; Fujisaka et al., 2018), environment (De Filippo et al., 2010; Yatsunenko et al., 2012), and antibiotic treatment (Reijnders et al., 2016; Liu X. et al., 2019), can affect the composition of the human gut microbiota, resulting in a highly dynamic ecosystem, yet only a few studies to date have focused on the interaction between exercise and gut microbiota (Clarke S. F. et al., 2014; Petersen et al., 2017). Still, these studies suggest that exercise may influence the gut microbial community at compositional and functional metabolic levels.

Recently, efforts have been engaged to decipher how exercise influences the human gut microbiome. For example, a comparison of the microbial communities of rugby players and healthy controls revealed that exercise increased bacterial diversity and was associated with differences in the metabolic capacity of gut microbiota (Clarke S. F. et al., 2014; Barton et al., 2018). Specifically, certain microbial metabolic pathways involved in carbohydrate metabolism and amino acid and short-chain fatty acids (SCFAs) synthesis were enriched in rugby players. Kulecka et al. (2020) showed that Polish endurance athletes have increased microbial diversity resulting from excessive training compared with the control group. Moreover, different classes of athletes within the same sport also presented with different gut microbiome profiles with regard to taxonomical and functional compositions, while these differences were correlated with physical performance (Petersen et al., 2017; Han et al., 2020). However, thus far, these previous investigations focused primarily on the microbial characteristics in professional athletes, and a considerable proportion of such studies did not report fecal metabolomics data. Considering the substantial inter-variability of microbiome features, the impact of exercise on the gut microbiome in soldiers remains elusive.

During training or combat, soldiers are commonly subjected to prolonged physical and emotional stress in a military environment that includes sustained physical exertion, sleep deprivation, exposure to environmental extremes, and less opportunities for dietary consumption and recovery (Lieberman et al., 2005). This combined stress could induce the release of catabolic hormones, inflammatory cytokines, and microbial molecules through the activation of the neuroendocrine system, ultimately altering the physiological homeostasis of soldiers (Morgan et al., 2015). Recent evidence has shown that alterations in gut microbial composition are closely associated with physical and emotional stress during exercise, apart from regulating skeletal muscle function (Clarke G. et al., 2014; Hawley, 2020). Specifically, the gut microbiota can respond to physical and emotional stress by modulating excitatory and inhibitory neurotransmitters and by promoting the release of hormones including neuropeptide Y and dopamine (Clarke G. et al., 2014; Holzer and Farzi, 2014). Given the diverse effects of the microbiota on host physiological function, it will be worthwhile to assess how the gut microbiota and fecal metabolites change in both elite and non-elite soldiers and determine whether any significant differences exist between the two groups. Understanding the role of the gut ecosystem in soldier performance is particularly interesting for soldiers who strive to improve their results and to reduce recovery time during training or combat. Furthermore, such information may be beneficial to human health.

To bridge such gaps, we conducted a cross-sectional 16S rRNA gene sequencing analysis of 80 fecal samples obtained from elite soldiers (ES, *n* = 40) and non-elite soldiers (N-ES, *n* = 40) to characterize gut microbiota features. Fecal metabolites are crucial in establishing host-microbiota cross-talk (Zierer et al., 2018). Combined microbial and metabolic analyses is a well-recognized approach for uncovering the taxonomic and functional signatures of the gut microbiome (Zheng et al., 2020). Thus, liquid chromatography-mass spectrometry (LC-MS) was performed for untargeted fecal metabolomics analysis. With the integration of multi-omics data, we characterized the patterns of altered microbiota and fecal metabolites, explored their reciprocal interactions, and further revealed how these alterations could modulate host metabolism. More importantly, a shortlist of microbes and metabolites was identified as potential biomarkers, facilitating the establishment of a random forest classifier to monitor the potential of ES with high accuracy. Collectively, our findings may provide insights for developing novel strategies to improve soldier performance based on microbiome-assisted approaches.

## Materials and Methods

### Study Design and Sample Collection

Male soldiers from the identical motorized infantry battalion, ≥18 years of age, with ≥2 years of military training, were eligible for this study if they did not present with major medical issues and did not consume antibiotics or any additional dietary supplementation, such as protein supplements, probiotics, and prebiotics, within the previous 6 months. The military training protocol followed by these soldiers was weekly designed in line with “Military Sports Training Outline” and included general physical training and specialized training for different armed branches. The general physical fitness assessment includes the performance of pull-ups, a 3-km run, a 30-m shuttle run, and sit-ups; the scoring criteria are divided into the following four classes: excellent (≥ 360), good (320–359), passed (240–319), and failed (< 240). Individuals who presented with excellent results in all four quarterly assessments during 2020 were thus categorized as ES (*n* = 40), while those who merely passed all tests were classified as N-ES (*n* = 40, Supplementary Table 1). All fecal samples were collected from January 10 to 12, 2021, according to previous methods (Santiago et al., 2014; Gratton et al., 2016). Briefly, an entire fecal sample was self-collected using the fecal collection kit (02544208; Thermo Fisher Scientific), placed immediately in an ice bag, and delivered to the lab within 3 h. Once received, the sample was manually homogenized on ice with a sterile stick within 1 h and divided into two 1.5 mL sterilized tubes (200 mg per tube) for storage at −80°C until further analysis.

The baseline characteristics of the soldiers are presented in Table 1. Body composition, including body and skeletal muscle mass indices and fat percentage, were measured with bioimpedance using the InbodyS10 device (InBody Co., Ltd., Seoul, South Korea). Quantitative diet evaluation was based on the 24-h diet recall method for three consecutive days. The other data, including duration and frequency of weekly physical efforts, fatigue states, and psychological factors, were collected using questionnaire surveys. To investigate the differences in characteristics between the two groups, we compared the anthropometric indicators, dietary factors, sport-related features, psychological states, ranks, and specialties, which showed that factor1 and factor2 of the fatigue assessment instrument (FAI) were significantly different (Table 1). All soldiers provided written informed consent before enrollment. This study was approved by the Ethics Committee of the Affiliated Jinling Hospital, Medical School of Nanjing University, Nanjing, China (2020NZGKJ-071).

**TABLE 1:** Baseline characteristics of subjects used in this study.

Parameters	ES (*n* = 40)	N-ES (*n* = 40)	*P*
**Anthropometric indicators**			
Age (year)	21.80 ± 1.59	21.25 ± 1.69	0.138*
Body weight(kg)	65.98 ± 6.07	67.30 ± 6.47	0.434^#^
BMI (kg/m^2^)	21.85 ± 1.22	21.45 ± 1.13	0.084^#^
SMI (kg/m^2^)	11.13 ± 0.95	10.98 ± 0.73	0.431*
ASMI(kg/m^2^)	9.14 ± 0.75	9.31 ± 0.56	0.096^#^
BFP(%)	6.78 ± 1.64	6.27 ± 1.95	0.169^#^
FMI(kg/m^2^)	1.49 ± 0.39	1.35 ± 0.43	0.089^#^
FFMI(kg/m^2^)	20.36 ± 1.10	20.10 ± 1.09	0.338^#^
**Dietary factors**			
Energy(kcal)	3354.74 ± 200.93	3430.89 ± 219.77	0.110*
Carbohydrates(g)	383.50 ± 35.36	396.91 ± 41.33	0.194^#^
Sucrose(g)	56.38 ± 6.93	58.70 ± 7.72	0.172^#^
Fiber(g)	23.88 ± 2.70	22.95 ± 2.84	0.139*
Protein (g)	163.77 ± 27.64	160.92 ± 26.61	0.640*
Fat(g)	129.52 ± 13.29	133.29 ± 13.19	0.207*
Saturated fatty acid(g)	43.94 ± 5.54	42.22 ± 4.74	0.140*
Monounsaturated fatty acid(g)	46.03 ± 7.23	47.47 ± 6.03	0.339*
Polyunsaturated fatty acid(g)	22.61 ± 3.38	23.39 ± 4.76	0.441^#^
Carbohydrates(%) of energy	45.73 ± 3.29	46.23 ± 3.15	0.490*
Protein (%) of energy	19.50 ± 2.94	18.77 ± 2.98	0.273*
Fat (%) of energy	34.77 ± 3.13	35.00 ± 3.12	0.742*
**Sport-related features**			
Impedance (Ω)	427.97 ± 34.03	436.58 ± 30.30	0.235*
Grip strength(kg)	48.17 ± 7.31	50.16 ± 6.89	0.215*
Exercise load (h/week)	18.63 ± 3.60	17.05 ± 3.40	0.336^#^
Training units per week	11.08 ± 1.51	10.50 ± 1.57	0.120^#^
**Fatigue assessment instrument**			
Factor1	3.092 ± 0.619	4.89 ± 1.27	<0.0001^#^
Factor2	3.82 ± 1.12	4.60 ± 1.27	0.007^#^
Factor3	5.02 ± 1.68	4.72 ± 1.64	0.289^#^
Factor4	6.51 ± 0.65	6.45 ± 0.71	0.747^#^
**Psychological states**			
SAS	35.60 ± 6.47	37.48 ± 5.80	0.176*
SDS	36.93 ± 4.74	35.65 ± 5.47	0.269*
**Military ranks**			
Corporal	26	20	0.369**^†^**
Sergeant	10	13	
Staff sergeant	4	7	
**Specialties**			
Infantry	24	27	0.401**^†^**
Artillery	9	10	
Scout	7	3	

BMI, body mass index; SMI, skeletal muscle mass index; ASMI, appendicular skeletal muscle mass index; BFP, body fat percentage; FMI, fat mass index; FFMI, fat-free mass index; SAS, Self-rating anxiety scale; SDS, Self-rating depression scale; Values are means ± standard deviation; *Student’s t-test; ^#^Mann–Whitney U-test; ^†^Pearson’s chi-squared test.

### DNA Isolation and 16S rRNA Gene Sequencing

DNA extraction from fecal samples was performed using the PowerSoil DNA Isolation Kit (12888-100; MO BIO Laboratories Inc., Carlsbad, CA, United States) according to the manufacturer’s instructions. The concentration of the extracted bacterial DNA was assessed using the Qubit 2.0 Fluorometer (Thermo Fisher Scientific, Waltham, MA, United States; Supplementary Table 2). The V3–V4 region of the 16S rRNA gene was amplified using the forward primer 5′-ACTCCTACGGGAGGCAGCA-3′ and the reverse primer 5′-GGACTACHVGGGTWTCTAAT-3′ combined with adapter and barcode sequences (Supplementary Table 3). The PCR products were purified using VAHTSTM DNA Clean Beads (N411-01; Vazyme Biotech Co., Ltd., Nanjing, China) and were quantified using the Quant-iT™ dsDNA HS Reagent (Q33232; Thermo Fisher Scientific). All sequencing reactions were performed using the Illumina NovaSeq 6000 v1.5 reagents (Illumina, San Diego, CA, United States) with 2 × 250 paired-end reads.

Subsequently, the 16S rRNA gene amplicons were processed to acquire high-quality sequences. The paired-end reads were merged using FLASH version 1.2.11^1^ (Magoc and Salzberg, 2011). Chimera sequences were removed using UCHIME version 8.1^2^ (Edgar et al., 2011). The data analysis, operational taxonomic unit (OTU) classification, and annotations of taxonomic information were achieved using Quantitative Insights Into Microbial Ecology version 2.0 (QIIME2,^3^ (Bolyen et al., 2019). OTUs were clustered using Uparse 10.0^4^ (Edgar, 2013) at the 97% identity level. Taxonomic annotations were obtained using the classify-sklearn algorithm^5^ and the Silva database (Release132,^6^ (Callahan et al., 2016). Alpha and beta diversity values were calculated using QIIME2. We selected Chao1, Shannon, Simpson, and phylogenetic diversity indices to evaluate the alpha diversity of microbial communities. For determining beta diversity, the gut microbiome similarity between different groups was investigated using principal coordinates analysis (PCoA) based on unweighted UniFrac, binary Jaccard, and weighted UniFrac distance metrics and visualized using R (version: 4.0.3). BugBase (Ward et al., 2017), an algorithm used for predicting biologically explicable phenotypes, was used to predict organism-level phenotype composition, including oxygen utilization, gram staining characteristics, and oxidative stress tolerance. Briefly, the OTU table and mapping file were uploaded selecting for output normalized OTU table, OTU contributions, and relative abundance plots for each microbiome phenotype.

### Co-occurrence Network Analysis

To describe the correlations among different genera, we constructed co-occurrence networks according to the 16S rRNA data (Wang et al., 2018). The microbial correlations in the ES and N-ES samples were investigated, respectively, in term of the relative abundance of each genus using Spearman’s correlation coefficient to establish the co-occurrence network. The cutoff of the absolute correlation was set at 0.35, and only significant correlations with a false discovery rate (FDR) <0.05 were visualized using Cytoscape version 3.8.1^7^ (Shannon et al., 2003). Further analysis was performed to measure the similarity between the two networks by shared correlations and closeness centrality of nodes. The edges with the same nodes in the two co-occurrence networks were defined as shared correlations. The closeness centrality indicates the importance of the nodes in each network. The results were illustrated using the Venn diagram and ggplot2 packages of R (version: 4.0.3).

### Correlation Analysis of Dominant Genera and Physical Phenotypes

To test whether microbiome changes were indicative of performance status, we conducted a correlation analysis between dominant genera and phenotypic scores (Bowerman et al., 2020). First, we identified the genera that presented with significant differences between the two groups. Thereafter, dominant genera with an at least 2-fold change between ES and N-ES cohorts were identified from the genera with significant differences. Finally, the correlations between significant dominant genera (average relative abundance >0.03%) and physical phenotypic scores, including physical fitness assessment mean scores and the FAI, were undertaken using Spearman’s correlations in R version 3.6.1 (psych package), and the matrix was produced using R version 3.6.1(corrplot package).

### Metabolite Extraction, Profiling, and Analysis

Fecal samples obtained from the ES and N-ES cohorts were subjected to metabolomic analysis based on the LC-MS method. Each 25-mg fecal sample was mixed with 1 mL extract solution (methanol: acetonitrile: water = 2: 2: 1) that included a standard mixture of L-leucine-5,5,5-d3 (0.004 mM), Betaine-(trimethyl-d9) hydrochloride (0.0002 mM), trimethylamine-d9 N-Oxide (0.001 mM), hippuric acid-d5 (0.0003 mM), [13C3]-L-(+)-sodium lactate (0.002 mM), and L-glutamic acid-13C5,15N (0.006 mM) for quality control. Subsequently, the mixtures were homogenized at 35 Hz for 4 min, sonicated for 5 min in an ice-water bath, and were then incubated for 1 h at −40°C. The samples were centrifuged at 12,000 rpm for 15 min at 4°C, and the resulting supernatant was transferred to a fresh glass vial for analysis using a UHPLC-QE Orbitrap/MS (Thermo Fisher Scientific) (Wang et al., 2014).

The LC-MS analysis was conducted according to a previous method (Li et al., 2017). Briefly, the analysis was conducted using the Vanquish UHPLC system (Thermo Fisher Scientific) with a UPLC BEH Amide column (2.1 mm × 100 mm, 1.7 μm) coupled to the Q Exactive HFX mass spectrometer (Orbitrap MS; Thermo Fisher Scientific). The mobile phase comprised ammonium acetate (25 mM) and ammonia hydroxide (25 mM) in water (pH 9.75) (A) and acetonitrile (B). Approximately 3 μL of the sample was injected at 4°C for analysis. The QE HFX mass spectrometer was used with the acquisition software (Xcalibur; Thermo Fisher Scientific) to acquire the full scan MS/MS spectra. The positive and negative spray voltages were 3.6 and 3.2 kV, respectively, the capillary temperature was 350°C, and the full MS and MS/MS resolution was set at 60,000 and 7500, respectively.

The raw data were converted to the mzXML format using ProteoWizard and processed using R package XCMS for peak detection, extraction, and integration (Smith et al., 2006). The metabolites were annotated with the featured peaks according to an in-house MS2 database (BiotreeDB). We removed the impurity peaks, and the identifications were performed in duplicate. For each dataset, the compounds present in more than 50% of the samples collected in this study were retained. The data on missing values were filled by half of the minimum value (Dunn et al., 2011). Thereafter, the internal standard normalization method was used for subsequent data analysis.

Principal component analysis (PCA) and orthogonal partial least-squares discriminant analysis (OPLS-DA) were applied to assess the differences in metabolic profiles between elite and non-elite soldiers using 14,182 metabolites detected from 80 fecal samples. The analysis was performed using the SIMCA16.0.2 software package (Sartorius Stedim Data Analytics AB, Umea, Sweden). The qualitative metabolites with variable important in projection (VIP) >1 and *P*-value <0.05 (Student’s *t*-test) were identified as significantly changed metabolites. Furthermore, commercial databases, including KEGG^8^ and MetaboAnalyst,^9^ were used for pathway enrichment analysis. The results were visualized using R version 4.0.3 (ggplot2 package) and Cytoscape version 3.8.1.

### Microbiome and Metabolome Data Integration

To determine the relationships between the microbiome and metabolome in soldiers, correlation analysis between dominant genera (average relative abundance >0.05%) and significantly different metabolites with >2.5-fold changes was performed using R version 3.6.1 (psych and corrplot package). Significant correlations, defined as absolute coefficient >0.6 and FDR <0.05, were included in the downstream analysis and were visualized using Cytoscape version 3.6.1.

### Biomarker Analysis and Random Forest Model Prediction

Linear discriminant analysis (LDA) effect size (LEfSe) (Segata et al., 2011) was conducted to identify differential taxonomical features between the two groups. Specifically, the *P*-values for the Wilcoxon rank-sum test and the logarithmic LDA score for distinct features were set at 0.05 and 3.5, respectively. The random forest model was established using the randomForest package in R to identify representative biomarkers. With respect to metabolic biomarkers, we selected two metabolites that demonstrated the highest fold changes in the most enriched metabolic pathway, according to a previous study (Guo et al., 2020). Thereafter, tenfold cross-validation was undertaken using a random forest model 50 times to quantify the discriminative performance of these biomarkers with average accuracy. Additionally, receiver operating characteristic analysis was conducted to evaluate the efficiency of possible cutoff values in the tests (Falony et al., 2016).

### Statistical Analysis

Statistical analysis was performed primarily using the R platform.^10^ The differences in taxa abundance and diversity indices were assessed using the Mann–Whitney *U*-test. All *P*-values have been corrected and presented as FDR using the Benjamini–Hochberg procedure (Guo and Bhaskara Rao, 2008). ANOSIM (Clarke, 1993) was performed using unweighted UniFrac, binary Jaccard, and weighted UniFrac distance metrics to evaluate the statistical significance of differences in the gut microbiota structures across the groups. Multivariate and univariate analyses were used to identify differential metabolites specifically defined as VIP >1 and *P* < 0.05, respectively (Student’s *t*-test). Microbiome and metabolome data have been presented as mean ± SD. Statistical significance was set at *P* < 0.05.

## Results

### Alterations of Gut Microbial Communities in Elite and Non-elite Soldiers

**FIGURE 1:**
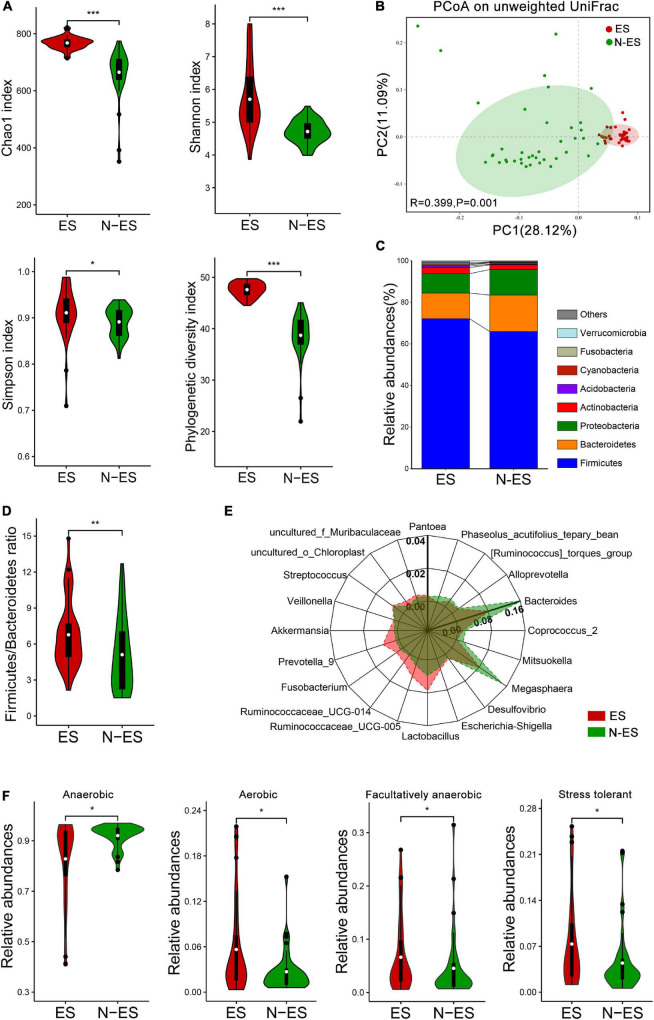
Characteristics of the gut microbiota in elite soldiers (ES) and non-elite soldiers (N-ES). **(A)** Comparison of Chao1, Shannon, Simpson, and phylogenetic diversity indices of microbial communities between the two groups. **(B)** Principal coordinates analysis (PCoA) of the gut microbiota based on unweighted UniFrac distance metrics for ES and N-ES. ANOSIM, *R* = 0.399, *P* = 0.001. **(C)** Bacterial proportions at the phylum level. **(D)** The values of *Firmicutes*/*Bacteroidetes* of ES showing significantly higher than that of the N-ES cohort. **(E)** Radar charts depict information on the discriminative microbiota between ES and N-ES cohorts at the genus level. The differential genera with mean relative abundances more than 0.2% of the total abundance are presented for clarity. Except for *Bacteroides* with the unique axis scale, the remaining genera have been presented using the same options for axes. **(F)** The estimate of phenotypic compositions in ES versus N-ES. *P < 0.05, **P < 0.01, ***P < 0.001, Mann–Whitney *U*-test with false discovery rate correction.

A total of 6,379,338 high-quality sequencing reads were identified across all fecal samples provided by elite and non-elite soldiers, ranging from 79,042 to 80,401 sequences (Supplementary Table 4). These sequences were clustered into 51,890 OTUs, ranging from 265 to 773 OTUs (Supplementary Table 4). Rarefaction curves suggested the achievement of high coverage (∼99%) for all samples, indicating sufficient sequencing depth for investigating gut microbiota and limited benefit of additional sampling (Supplementary Figure 1A and Supplementary Table 4). Analysis of the data obtained herein suggested that the microbial diversity and richness indices were significantly higher in the ES group than those in the N-ES group (Figure 1A). PCoA was performed to explore the similarity of the overall microbial communities in the two cohorts based on unweighted UniFrac (Figure 1B), binary Jaccard (Supplementary Figure 1B), and weighted UniFrac distance metrics (Supplementary Figure 1C). The analysis revealed that the microbial compositions of the ES cohort clusters were more homogeneous and significantly distinct from those of the N-ES cohort.

At the phylum level, the four most abundant phyla identified in soldiers were *Firmicutes*, *Bacteroidetes*, *Proteobacteria*, and *Actinobacteria* (Figure 1C), and this finding was consistent with that reported within the Chinese population (Liu H. et al., 2019). Thereafter, comparisons of the relative abundances of these phyla revealed that elite soldiers harbored significantly greater proportions of 10 taxa than N-ES, while the proportion of only 1 taxon, *Bacteroidetes*, was significantly decreased in elite soldiers (Supplementary Figure 2A). Recent studies have shown that the *Firmicutes*/*Bacteroidetes* (F/B) ratio is closely associated with increased physical performance (Durk et al., 2019). Hence, F/B values were calculated, and the ES cohort exhibited significantly higher F/B values (Figure 1D). At the genus level, 226 discriminative organisms were identified between the two groups (Supplementary Table 5). Specifically, we found that *Ruminococcaceae_UCG-005*, *Lactobacillus*, *Prevotella_9*, and *Veillonella* were enriched in the ES cohort, while *Bacteroides*, *Megasphaera*, and *Coprococcus_2* were dominant in the N-ES cohort (Figure 1E and Supplementary Figure 2B). Additionally, the hierarchical heatmap demonstrated that the top 30 significantly different genera detected across all samples presented different patterns between the ES and N-ES groups (Supplementary Figure 2C).

Based on the taxonomical composition, the differences in the phenotypic compositions of ES and N-ES cohorts were investigated using Bugbase (Ward et al., 2017). Significantly greater relative abundances of aerobic, facultative anaerobic, and oxidative stress tolerant microbiota were observed in ES relative to N-ES, while anaerobic microbiota were significantly less abundant in ES (Figure 1F). However, there were no significant differences in the proportions of gram-positive and gram-negative bacteria between the two groups.

**FIGURE 2:**
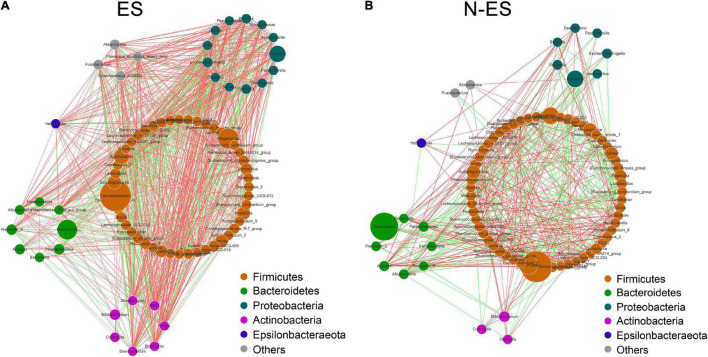
The co-occurrence networks constructed based on relative abundances of genera in each group. **(A,B)** Genus–genus co-occurrence networks of ES **(A)** and N-ES **(B)** according to the Spearman correlation algorithms. Each node indicates a bacterial genus. The node size represents the relative abundance of these genera. Edges between nodes stand for positive (light red) or negative (light green) correlation, and the edge thickness indicates the Spearman coefficient.

To gain insights into potential relationships among bacterial genera within the microbial communities from an ecological perspective, the genus–genus co-occurrence networks of each group were constructed based on Spearman correlations. Within the two co-occurrence networks, the differential genera belonged to *Firmicutes*, *Bacteroidetes*, *Proteobacteria*, *Actinobacteria*, and *Epsilonbacteraeota* (Figures 2A,B). The ES group presented with a more complicated co-expression network with strong positive correlation among genera (Figure 2A). Conversely, the correlation between microbes in the N-ES group was distinctly weak (Figure 2B). To quantify such differences, we determined the number of edges (correlations) and the closeness centrality of nodes (genera) in the two networks. The Venn diagram displayed 96 edges shared by both groups, while 873 and 327 edges were unique to the ES and N-ES groups, respectively (Supplementary Figure 3A). Moreover, the closeness centrality of shared genera also exhibited distinct differences between the two groups (Supplementary Figure 3B). Taken together, the above-mentioned analyses indicated that the microbial interactions in the ES cohort exhibited alterations compared with those in the N-ES cohort, which might generate disparate synergistic and niche-related relationships and partly account for differences in the physical performance of soldiers.

### Gut Microbiome Changes Reflecting Performance Status

**FIGURE 3:**
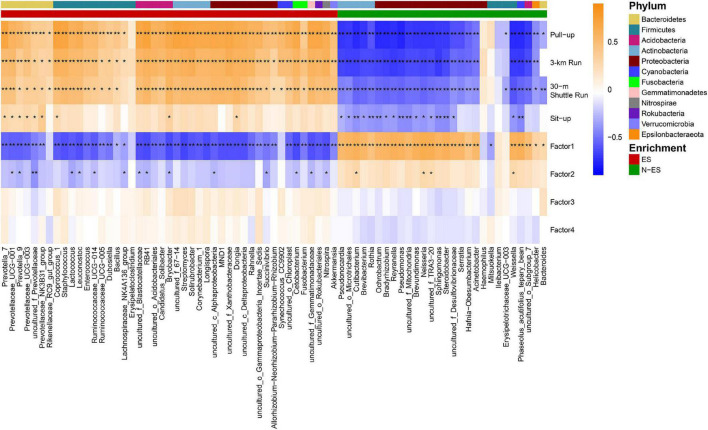
Correlations between the members of the gut microbiome with physical phenotypes. Spearman’s rank correlations were calculated between dominant genera (> 0.03%) and phenotypic scores. Higher taxonomy of genera (phylum) with enrichment in either ES or N-ES samples have been indicated by the colored bar at the top portion. The correlation effect is shown by a color gradient from blue (negative correlation) to orange (positive correlation). Black stars within heatmap boxes represent significant results (*P < 0.05, **P < 0.001, Student’s *t*-test with false discovery rate correction for multiple comparisons).

To explore whether the differences in the microbiome signature were related to performance status, we conducted Spearman’s rank correlation analysis between dominant genera (average relative abundance >0.03%) and the phenotypic scores of all soldiers (Figure 3). We found that the ES group could be characterized by 45 enriched dominant genera mainly belonging to *Bacteroidetes* and *Firmicutes* (18/45), and almost all members of the *Bacteroidetes* (6/7), such as *Prevotella_7*, *Prevotella_9*, and *Prevotellaceae_UCG-003*, were positively associated with the mean score of each physical fitness assessment item. Conversely, the dominant microbiota enriched in the N-ES group mainly belonged to *Actinobacteria* and *Proteobacteria* (20/28), and most genera (16/20), including *Rothia*, *Pseudomonas*, and *Sphingomonas*, exhibited negative correlations with four physical fitness assessment results. Alternatively, four distinct dimensions of fatigue were identified in the FAI. The first factor could be perceived as a quantitative indicator of the overall fatigue used to measure fatigue severity, the second factor was indicative of the sensitivity of fatigue to specific circumstances, the third factor addressed possible consequences of fatigue, and the fourth factor indicated the response of fatigue to rest/sleep (Schwartz et al., 1993). In this study, the enriched genera in the ES cohort mostly exhibited strong negative correlations with Factor1, while a considerable proportion of the enriched genera in the N-ES cohort and Factor1 were strongly and positively correlated. Overall, these findings support an association between the gut microbiome and soldier performance status, aiding the identification of genera associated with an enhanced exercise phenotype. However, further investigation of longitudinal samples is needed to detect long-term changes in microbial composition.

### Metabolic Pattern Differences Between ES and N-ES Groups

**FIGURE 4:**
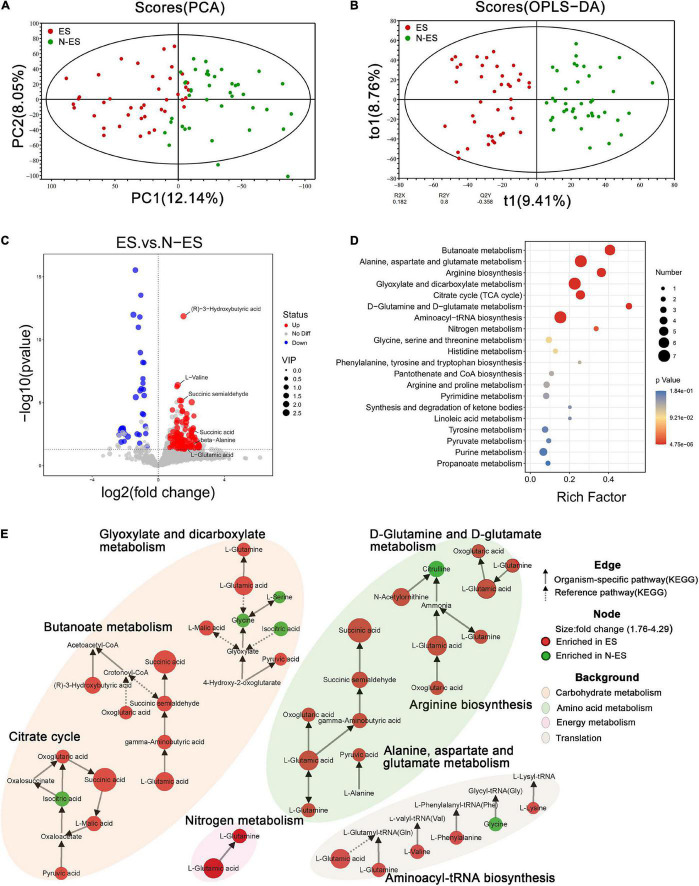
Discrepant metabolic patterns in elite soldiers (ES) and non-elite soldiers (N-ES). **(A,B)** The clustering analyses of principal component analysis (PCA) and orthogonal partial least-squares discriminant analysis (OPLS-DA). **(C)** Volcano plots showing the *P*-value (*y*-axis), fold-change (*x*-axis), and variable important in projection (VIP, node size) of the qualitative metabolites identified in the metabolomics analysis. The color of the circle represents differential metabolites (VIP >1, *P* < 0.05 Student’s *t*-test) with enrichment (red) or depletion (blue) in ES group. **(D)** Bubble diagrams showing the top 20 enriched Kyoto Encyclopedia of Genes and Genomes (KEGG) pathways for the differential metabolites. Abscissa variations indicate the degree of enrichment (Rich factor), and the vertical axis shows the KEGG pathway information. The color scale indicates significance levels, and the node size represents the number of discriminative metabolites in the mapping pathway. **(E)** The significantly enriched KEGG pathway (*P* < 0.05 hypergeometric test) of the differential metabolites. Identified metabolites are represented by nodes with the size reflecting fold change. Each node is labeled with red (enriched in ES cohort) or green (enriched in N-ES cohort). The solid and dotted arrows symbolize organism-specific and reference pathways, respectively, according to the KEGG database. The disparate color shadows highlight the pathway at KEGG level 2.

The fecal metabolome is acknowledged as a direct functional readout of the gut microbiome (Zierer et al., 2018). Hence, we explored the host metabolic signatures in the same samples and detected 14,182 metabolites, of which 1,370 had known chemical identity, including 1,092 in the positive and 278 in the negative ion mode. The fecal samples obtained from different cohorts could be completely separated according to PCA and OPLS-DA (Figures 4A,B), suggesting distinct differences in the overall metabolic phenotypes.

Subsequently, 133 discriminative metabolites were identified between the ES and N-ES groups (Supplementary Table 6). Compared with the N-ES group, elite soldiers could be characterized by 97 enriched metabolites and 36 depleted metabolites (Figure 4C), among which the 30 most significantly different metabolites exhibited a dissimilar metabolic mode between the two groups (Supplementary Figure 4). The enrichment analysis of these altered metabolites identified eight significantly enriched metabolic pathways, including butanoate metabolism, alanine, aspartate, and glutamate metabolism, and arginine biosynthesis (Figure 4D), which were primarily associated with carbohydrate, amino acid, and energy metabolism (Figure 4E). Notably, most metabolites clustered in these pathways, such as (R)-3-hydroxybutyric acid, succinic acid, and pyruvic acid, were enriched in the ES group, while the N-ES group exhibited enrichment in only four metabolites, L-serine, glycine, citrulline, and isocitric acid (Figure 4E). Studies have shown that exercise capacity is closely correlated with the energy-related metabolic pathways performed by the gut microbiota, especially carbohydrate metabolism, because polysaccharides and monosaccharides are the primary substrates that are oxidized to meet energy demands during prolonged subjection to submaximal exercise (van Loon et al., 2001; Mach and Fuster-Botella, 2017). Therefore, our results revealed that the ES group exhibited a more energetically efficient metabolic phenotype, which seemed to be crucially important for enhancing performance.

### Integration of the Microbiome and Metabolomes

**FIGURE 5:**
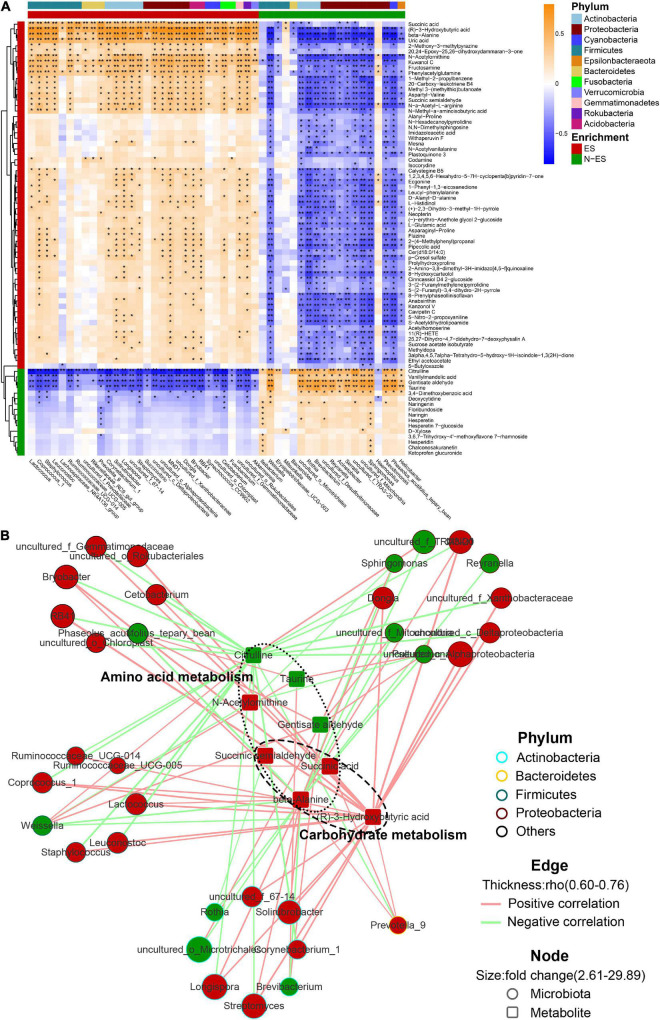
The multi-omics correlation analysis of the microbiome and metabolome. **(A)** Correlations between dominant genera (> 0.05%) and metabolites with >2.5-fold changes between ES and N-ES, VIP >1, and *P* < 0.05 (Student’s *t*-test). Enrichment in either group and higher levels of taxonomy (phylum) are indicated by colored bars at the left and top of the heatmap. Significant correlations are denoted by black stars (*P < 0.05, **P < 0.001, Student’s *t*-test with false discovery rate adjustment for multiple comparisons). **(B)** Network profiles of the correlations with absolute coefficients higher than 0.6. The node shape represents the components involved in this study (circle: microbiota; round rectangle: metabolite). In the network, these metabolites mainly belong to carbohydrate and amino acid metabolism. The colors of the border of circles indicate different phyla. The node size indicates the fold change, and the color reflects higher abundance in either ES (red) or N-ES (green) samples. Edges between nodes stands for Spearman’s positive (light red) or negative (light green) correlation; the edge thickness denotes the range of the absolute coefficients.

Next, we investigated the potential relationships between the abundances of dominant genera (> 0.05%) and fecal metabolites. Overall, the multi-omics correlation analysis revealed strong and broad relationships, many of which involved microbes and metabolites enriched in the ES group (Figure 5A). Seventy-nine significant associations with absolute Spearman’s coefficients higher than 0.6 were found (Figure 5B). Within this network, the scattered genera were principally assigned to *Actinobacteria*, *Bacteroidetes*, *Firmicutes*, and *Proteobacteria*. The enriched microbiota in the ES cohort, such as *Prevotella_9*, *Lactococcus*, and *Ruminococcaceae_UCG-005*, were positively correlated with many fecal metabolites, including beta-alanine, succinic acid, and (R)-3-hydroxybutyric acid, which contributed to carbohydrate and amino acid metabolism. However, the microbiota enriched in the N-ES cohort, such as *Brevibacterium* and *Weissella*, presented numerous negative correlations with the aforementioned metabolites and exhibited positive correlations with only three metabolites associated with amino acid metabolism, citrulline, taurine, and gentisate aldehyde. These findings illustrated that diverse microbes dominated in different groups, and this could be the driving force for the differences in metabolic phenotypes.

### Combinatorial Biomarkers for Distinguishing Between ES and N-ES

**FIGURE 6:**
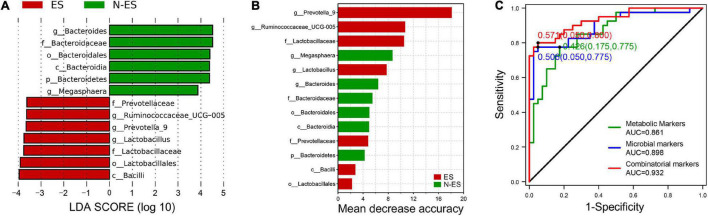
Multiple markers obtained for monitoring the potential of soldiers. **(A)** Microbial biomarkers obtained for identification of ES and N-ES cohorts. **(B)** The order of importance for these variables. The bar colors indicate enrichment in ES (red shades) or N-ES (green shades). **(C)** Classification performance of the random forest models based on metabolic markers (green), microbial markers (blue), and combinatorial markers (red) for ES and N-ES cohorts.

To further explore gut microbial community features at the taxonomic level, LEfSe was performed to identify microbial markers in each cohort. We obtained data on 13 taxa with LAD score >3.5, seven of which were more abundant in the ES cohort, including *Bacilli*, *Lactobacillales*, and *Lactobacillaceae* (Figure 6A). The random forest model was used to evaluate the feature importance and to identify the representative variations that could describe the most considerable deviations between the two cohorts (Yang et al., 2020). As shown in Figure 6B, the genera *Prevotella_9* and *Ruminococcaceae_UCG-005* were identified as biomarkers for the ES cohort. At the metabolic level, considering that the most enriched pathway was butanoate metabolism with a 4.3-fold change in the level of succinic acid and a 3.4-fold change in the level of L-glutamic acid (Figures 4D,E), we selected these two metabolites as biomarkers for the ES cohort according to a previously reported method (Guo et al., 2020).

To exploit the potential value of identified biomarkers for monitoring the potential of soldiers, 10-fold cross-validation was performed using a random forest model with 50-time repeats. The results showed that, based on the microbial biomarkers, the area under the curve (AUC) and average accuracy obtained for distinguishing between ES and N-ES samples were 0.898 and 85.48% (81.25–88.75%, Figure 6C), respectively. Similarly, based on the metabolic biomarkers, the AUC and average accuracy for identifying ES from N-ES samples were 0.861 and 82.43% (77.50–86.25%, Figure 6C), respectively. Nevertheless, we discovered that a combinatorial marker panel of these four biomarkers enabled the detection of ES samples from N-ES samples with high classification power (AUC: 0.932; average accuracy: 88.88%, ranging from 85 to 92.5%; Figure 6C), resulting in a more robust discriminative performance than that of microbial biomarkers or metabolic biomarkers alone. In summary, the gut microbiome and metabolome signatures in elite soldiers differ significantly from those of non-elite soldiers. Our findings may have potential applicability in the prediction of the potential of elite soldiers from a group of soldiers. However, further research is warranted to clarify the direct causation or correlation between these biomarkers and enhanced exercise performance using animal models.

## Discussion

In the present study, we first outlined the landscapes of microbial and metabolic signatures and their interactions established in the gut ecosystem of Chinese soldiers under conditions of sustained military training. The microbial communities of ES significantly differ from those of N-ES in terms of both diversity and the presence of several taxa. The enrichment of carbohydrate and amino acid metabolism was the hallmark of the ES gut ecosystem. Moreover, an accurate classifier was established based on a combinatorial marker panel, emphasizing considerable potential in distinguishing candidate elite soldiers from a group of soldiers. Since changes in the microbiome and metabolome correlated with performance status, our findings might lay the foundation for the development of performance-enhancing strategies by targeting specific bacteria that could beneficially influence metabolic efficiency.

Previous studies consistently reported that physical exercise could contribute to higher microbial diversity and alterations in microbial composition (Clarke S. F. et al., 2014; Han et al., 2020; Kulecka et al., 2020). Microbial diversity correlates with higher peak oxygen uptake (VO_2_ max/kg), which is crucial for exercise capacity (Estaki et al., 2016). In this study, the microbial richness and diversity of ES were significantly higher than those of N-ES. The more diverse microbiota can promote ecological stability and resist the effect of harmful environmental factors (Sommer et al., 2017). The proportions of certain microbial taxa were also altered between the ES and N-ES groups. The F/B ratio is usually used to characterize the general composition of gut microbiota. ES exhibited an elevated F/B ratio in accordance with previous reports on elite athletes (Han et al., 2020). Recently, accumulating evidence has demonstrated that the F/B ratio positively correlates with fecal total SCFAs (Mariat et al., 2009; Fernandes et al., 2014), particularly butyrate (Yamamura et al., 2020), and peak oxygen uptake (VO_2_ max/kg) (Durk et al., 2019). Compared with the N-ES group, ES presented a significantly higher abundance of the genera *Ruminococcaceae_UCG-005*, *Prevotella_9*, *Veillonella*, and *Akkermansia*. Members of these genera have been reported to produce SCFAs (Krautkramer et al., 2021) previously associated with numerous health benefits, including the promotion of skeletal muscle strength and endurance capacity (Morrison and Preston, 2016; Frampton et al., 2020). This finding is consistent with a recent study which has confirmed that the exercise-enhancing properties of *Veilonella atypica* are realized *via* its metabolic conversion of lactate into propionate (Scheiman et al., 2019). Hence, we speculate that the changes in the microbial composition of elite soldiers are conducive to increased SCFAs production, at least partly accounting for the improvement in their physical performance.

Additionally, mounting evidence has shown that microbial composition can also affect the responses to inflammatory and oxidative stress (Przewlocka et al., 2020). During physical training, there is an overproduction of reactive oxygen species and occurrence of low-grade systemic inflammation, leading to lipid and protein peroxidation and muscle cell metabolism disorders, which together ultimately disrupt muscle function (Merry and Ristow, 2016; Karl et al., 2017). The ES presented with an overrepresentation of *Lactobacillus*, which might contribute to inflammation reduction by stimulating the secretion of anti-inflammatory cytokines such as TGF-β, IL-10, and tryptophan-2,3-dioxygenase, and might aid oxidative stress reduction by elevating superoxide dismutase activity and intestinal glutathione levels (Przewlocka et al., 2020). Consistently, the proportion of oxidative stress tolerant microbiota was significantly higher in ES. Furthermore, *Akkermansia*, which has been reported to improve metabolic health (Dao et al., 2016), was also enriched in the ES cohort. *Akkermansia muciniphila* can help strengthen the integrity of the intestinal epithelial cell layer (Reunanen et al., 2015) and increase the thickness of the mucus layer by stimulating mucin production (Shin et al., 2014), contributing to reduced microbial translocation and inflammation. Moreover, the elevated abundance of *Prevotella_9* in the ES cohort, consistent with the results reported by Petersen et al. (2017), may result in the exertion of mucosal protective and immunoregulatory effects through the binding of its metabolite (succinic acid) to GPR91 on the surface of dendritic cells (Rubic et al., 2008; Kovatcheva-Datchary et al., 2015). Combined with the observed associations between dominant genera (> 0.03%) and exercise phenotypes, it can be speculated that these genera may mediate beneficial effects on the intestinal barrier, endotoxin translocation, and immune modulation, which may combine to prevent or mitigate the adverse effects of inflammatory and oxidative stress on skeletal muscle, thereby positively influencing exercise performance.

Gut microbiota can significantly shape the fecal metabolome. In our study, this viewpoint was also confirmed *via* metabolic function and multi-omics correlation analyses. In the metabolic network, all altered fecal metabolites were primarily involved in carbohydrate and amino acid metabolism. Consistently, fecal metabolites, substantially correlated with dominant microbiota (> 0.05%), were also mapped into these two categories of metabolic pathways. A recent study revealed the distinct alterations in microbial functional profiles characterized by a higher proportion of ATP, carbohydrate, and amino acid metabolism in elite athletes (Han et al., 2020). In line with these results, we found that most differential metabolites associated with carbohydrate and amino acid metabolism, especially succinic acid, were more abundant in the ES group than those in the N-ES group. On one hand, succinic acid, mainly produced by *Prevotella* (Kovatcheva-Datchary et al., 2015), is considered an SCFA precursor, which can be converted to methylmalonyl-CoA and later to propionate (Koh et al., 2016) that have well-established effects on improving exercise capacity (Scheiman et al., 2019). On the other hand, succinic acid is identified as a signaling molecule for mediating muscle exercise adaptations, including muscle innervation, extracellular matrix remodeling, and increased strength (Reddy et al., 2020). Beta-alanine, another important metabolite, acts as the rate-limiting precursor for synthesizing creatine that plays a crucial role as calcium regulator in skeletal muscle (Boldyrev et al., 2013). Elevated muscle carnosine concentrations are associated with performance-enhancing effects in high-intensity exercise (Blancquaert et al., 2017). Additionally, arginine biosynthesis is critically important for elite soldiers, which may be partly attributed to arginine increasing peroxisome proliferator-activated receptor-gamma coactivator 1α (PGC1α) expression (Chen et al., 2018). PGC1α serves as the master regulator of mitochondrial biogenesis and skeletal muscle fiber type, and therefore its upregulation may help promote an oxidative skeletal muscle phenotype, consequently improving exercise capacity (Chen et al., 2018; Kamei et al., 2020). Simultaneously, PGC1α-mediated NO production may increase blood flow through the skeletal muscle (Kamei et al., 2020). However, the impact of arginine on muscle growth seems to be limited here, owing to a non-significant difference in skeletal muscle mass index between ES and N-ES groups. By integrating these findings, we speculate that the microbially driven metabolic signatures of the ES cohort exert critical biological significance for physical performance.

As the present investigation is an exploratory study, several limitations of the data presented should be noted, especially concerning the cross-sectional design and lack of a matching non-soldier cohort. Moreover, to exclude potentially confounding effects, we used well-matched fecal samples to explore the molecular signatures inherent in elite soldiers. Of course, it should not be ignored that a 3-day diet may have a short and limited effect on the gut microbiome. Once the dietary habits or training intensity change, the gut microbiome and metabolome of soldiers exhibit responses in kind and undergo alterations. Hence, in future studies, we aim to perform a long-term follow-up analysis and to recruit more subjects, including healthy non-soldier and validation cohorts, to verify the stability of our findings as well as the generalizability of the identified biomarkers. Finally, blood metabolomic analysis may be favorable for gaining a deeper understanding of the mechanism by which the gut microbiome modulates host metabolism in soldiers.

## Conclusion

Using multi-omics data, we have provided evidence that ES can be characterized by the enrichment of SCFAs-producing bacteria and oxidative stress tolerant microbiota, as well as by more abundant metabolites associated with carbohydrate and amino acid metabolism. These alterations exert a synergistic effect on host metabolism, which may be strongly implicated in the enhanced physical phenotype. Furthermore, based on the combinatorial markers, we constructed a classifier that enabled effective distinction between elite and non-elite soldiers. Taken together, our findings provide new directions for developing novel performance-enhancing strategies for soldiers by targeting specific microbiota that drive more energetically efficient metabolic patterns.

## Data Availability Statement

The datasets presented in this study can be found in online repositories. The names of the repository/repositories and accession number(s) can be found below: https://www.osf.io/htvf8.

## Ethics Statement

The studies involving human participants were reviewed and approved by the Ethics Committee of the Affiliated Jinling Hospital, Medical School of Nanjing University. The patients/participants provided their written informed consent to participate in this study.

## Author Contributions

XW designed the study. YS, PW, and DZ collected the fecal samples. YS, LH, LZ, and XG took responsibility for data analysis. YS, GM, and SW prepared the figures. YS, PW, and XW wrote the draft of the manuscript. All authors revised the manuscript for important intellectual content.

## Conflict of Interest

The authors declare that the research was conducted in the absence of any commercial or financial relationships that could be construed as a potential conflict of interest.

## Publisher’s Note

All claims expressed in this article are solely those of the authors and do not necessarily represent those of their affiliated organizations, or those of the publisher, the editors and the reviewers. Any product that may be evaluated in this article, or claim that may be made by its manufacturer, is not guaranteed or endorsed by the publisher.

## Footnotes

1
http://ccb.jhu.edu/software/FLASH

2
http://www.drive5.com/usearch/manual/uchime_algo.html

3
https://qiime2.org/

4
http://drive5.com/uparse

5
https://github.com/QIIME2/q2-feature-classifier

6
http://www.arb-silva.de

7
http://www.cytoscape.org

8
http://www.genome.jp/kegg/

9
http://www.metaboanalyst.ca/

10
http://www.r-project.org/
