# Supplementary material for: Multi-Omics Analyses Characterize the Gut Microbiome and Metabolome Signatures of Soldiers Under Sustained Military Training
Source: Front Microbiol. 2022 Mar 25;13:827071. doi: 10.3389/fmicb.2022.827071 (PMC8990768; doi:10.3389/fmicb.2022.827071)
Supplement: Supplementary file 2 [file Data_Sheet_2.PDF]

Supplementary Material

1 Supplementary Figures

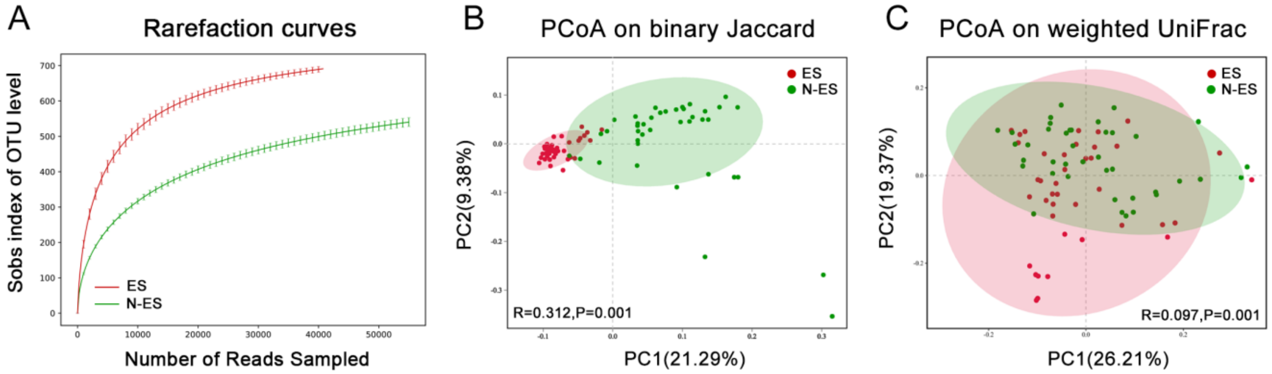

Figure S1. Gut microbiota analysis in elite and non-elite soldiers. (A) Rarefaction curves indicating the achievement of high coverage (~99%) for all fecal samples. Sobs index represents the observed number of OTUs. Principal coordinates analysis (PCoA) of the gut microbiota based on the binary Jaccard **(B)** and weighted UniFrac distance metrics **(C)**.

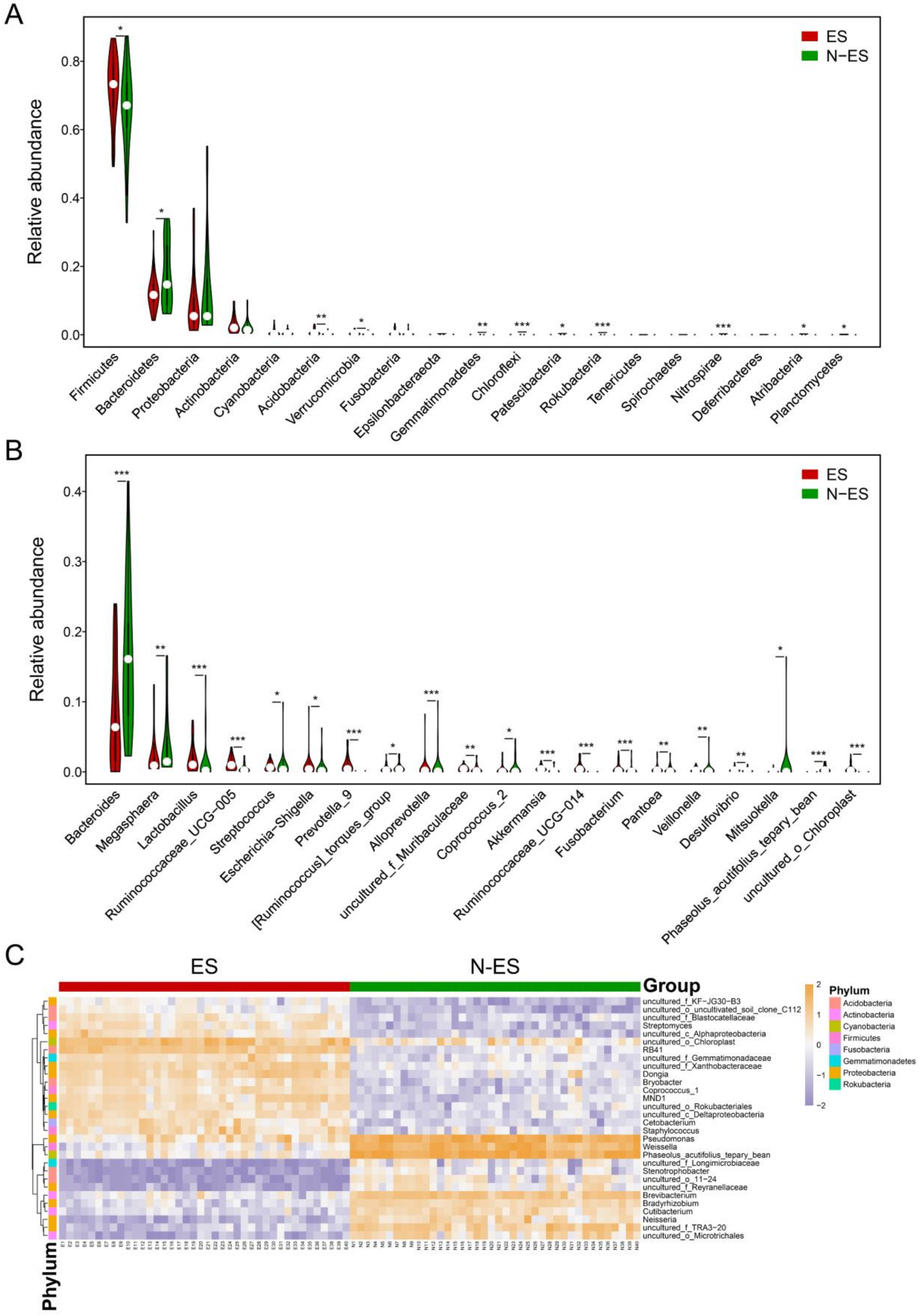
A
Relative abundance
0.0
0.2
0.4
0.6
0.8
ES
N−ES
Firmicutes
Bacteroidetes
Proteobacteria
Actinobacteria
Cyanobacteria
Acidobacteria
Verrucomicrobia
Fusobacteria
Epsilonbacteraeota
Gemmatimonadetes
Chloroflexi
Patescibacteria
Rokubacteria
Tenericutes
Spirochaetes
Nitrospirae
Deferribacteres
Atribacteria
Planctomycetes
B
Relative abundance
0.0
0.1
0.2
0.3
0.4
ES
N−ES
Bacteroides
Megasphaera
Lactobacillus
Ruminococcaceae_UCG−005
Streptococcus
Escherichia−Shigella
Prevotella_9
[Ruminococcus]_torques_group
Alloprevotella
uncultured_f_Muribaculaceae
Coprococcus_2
Akkermansia
Ruminococcaceae_UCG−014
Fusobacterium
Pantoea
Veillonella
Desulfovibrio
Mitsuokella
Phaseolus_acutifolius_tepary_bean
uncultured_o_Chloroplast
C
ES
N-ES
Group
uncultured_f_KF−JG30−B3
uncultured_o_uncultivated_soil_clone_C112
uncultured_f_Blastocatellaceae
Streptomyces
uncultured_c_Alphaproteobacteria
uncultured_o_Chloroplast
RB41
uncultured_f_Gemmatimonadaceae
uncultured_f_Xanthobacteraceae
Dongia
Bryobacter
Coprococcus_1
MND1
uncultured_o_Rokubacteriales
uncultured_c_Deltaproteobacteria
Cetobacterium
Staphylococcus
Pseudomonas
Weissella
Phaseolus_acutifolius_tepary_bean
uncultured_f_Longimicrobiaceae
Stenotrophobacter
uncultured_o_11−24
uncultured_f_Reyranellaceae
Brevibacterium
Bradyrhizobium
Cutibacterium
Neisseria
uncultured_f_TRA3−20
uncultured_o_Microtrichales
2
1
0
−1
−2
Phylum
Acidobacteria
Actinobacteria
Cyanobacteria
Firmicutes
Fusobacteria
Gemmatimonadetes
Proteobacteria
Rokubacteria
Phylum

Figure S2. **Differential taxonomical composition in the gut microbiome of soldiers. (A)** The taxonomical composition for ES and N-ES cohorts at the phylum level. **(B)** The 20 most abundant differential genera across ES and N-ES groups. **(C)** Heatmap illustrating the top 30 significantly different genera detected in the two groups. The sample locations and hierarchical cluster analysis grouping genera are shown by the colored bar at the top and on the left, respectively. The color gradient indicates the normalized values of relative abundances obtained via centering and log 10 transformation.

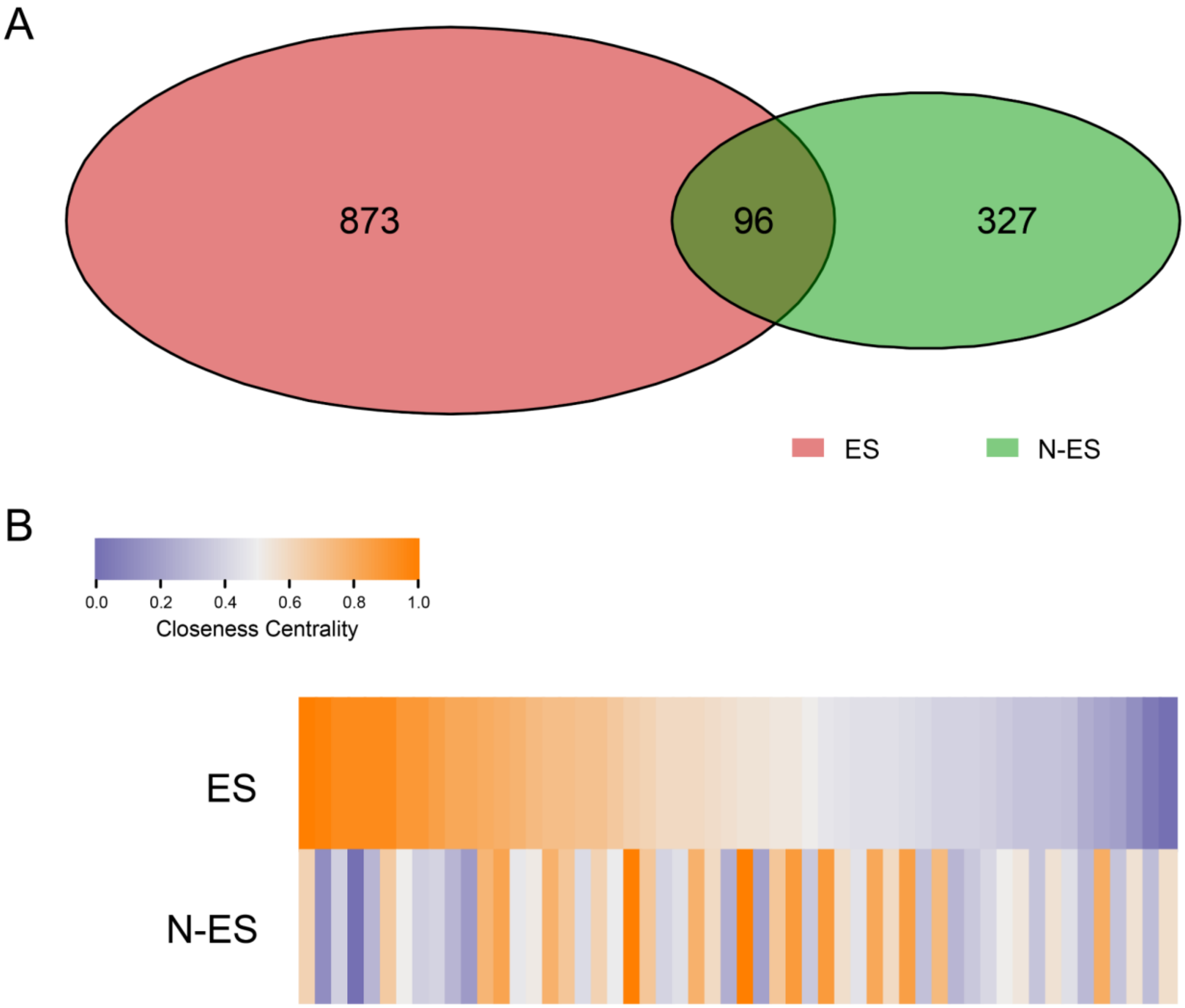

Figure S3. The differences of genera co-occurrence networks between ES and N-ES cohorts. The quantity of shared and unique edges **(A)** and closeness centralities of shared nodes **(B)** in ES and N-ES co-occurrence networks have been indicated.

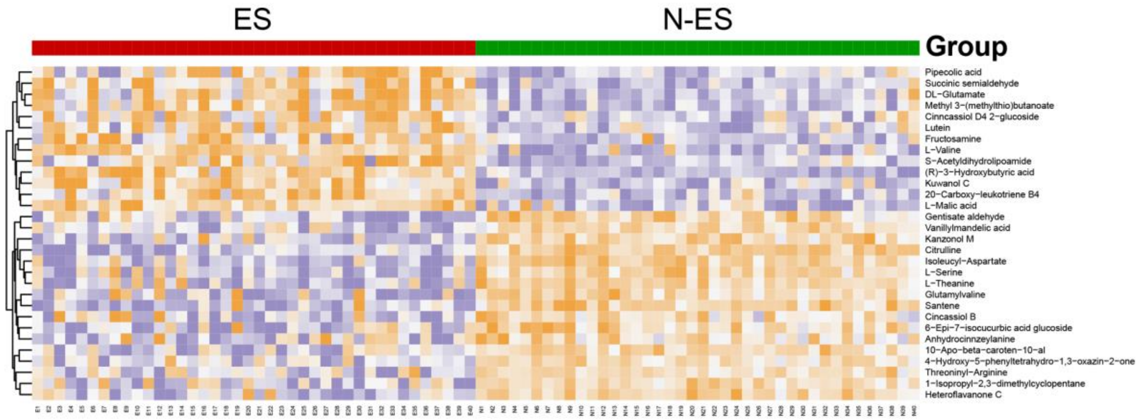

Figure S4. Heatmap of the 30 most significantly different metabolites across ES and N-ES groups. The sample grouping is shown at the top of the plot. The relative quantifications of metabolites have been centered using log 10 transformation prior to analysis.

2 Supplementary Tables

Table S1. General physical fitness assessment scores of soldiers.

Parameters	ES (n=40)	N-ES (n=40)	P
Pull-up			
First quarter	92.93±9.62	67.63±10.13	< 0.0001#
Second quarter	90.85±9.78	67.63±10.00	< 0.0001#
Third quarter	88.10±9.42	65.25±6.69	< 0.0001#
Fourth quarter	94.73±10.96	66.50±9.49	< 0.0001#
Mean	91.65±8.04	66.63±7.71	< 0.0001#
3-km Run			
First quarter	95.36±8.40	75.76±10.20	< 0.0001#
Second quarter	95.85±8.21	70.15±11.36	< 0.0001#
Third quarter	95.6±9.03	70.40±11.79	< 0.0001#
Fourth quarter	93.56±9.77	71.28±9.45	< 0.0001#
Mean	95.10±7.62	71.90±9.10	< 0.0001#
30-m Shuttle Run			
First quarter	90.90±7.10	70.38±12.06	< 0.0001#
Second quarter	93.10±8.22	73.75±15.43	< 0.0001#
Third quarter	94.38±6.82	75.50±15.68	< 0.0001#
Fourth quarter	92.83±9.90	72.88±15.31	< 0.0001#
Mean	92.80±6.48	73.13±13.62	< 0.0001#
Sit-up			
First quarter	97.25±8.24	86.75±10.28	< 0.0001#
Second quarter	97.50±8.560	89.03±10.66	< 0.0001#
Third quarter	98.43±8.39	88.75±8.44	< 0.0001*
Fourth quarter	95.13±10.87	87.58±10.48	0.002#
Mean	97.08±8.23	88.03±8.46	< 0.0001#
Total			
First quarter	376.45±17.61	300.53± 17.61	< 0.0001#
Second quarter	377.30±20.57	300.55±20.90	< 0.0001#
Third quarter	376.50±19.10	299.90±21.04	< 0.0001#
Fourth quarter	376.25±19.28	298.23±19.58	< 0.0001#
Mean	376.63±17.78	299.80±17.97	< 0.0001#

Values are means ± standard deviation; * Student's *t*-test; # Mann–Whitney U-test.

Table S2. The quality assessment of bacterial DNA.

Sample ID	OD260/280	OD260/230	Status	Group
E1	2.1	1.84	Normal	ES
E2	2.09	2	Normal	ES
E3	2.23	0.72	Normal	ES
E4	2.13	1.92	Normal	ES
E5	2.11	2.04	Normal	ES
E6	2.09	1.93	Normal	ES
E7	2.13	1.89	Normal	ES
E8	2.06	1.76	Normal	ES
E9	2.13	1.88	Normal	ES
E10	2.11	1.6	Normal	ES
E11	2.03	1.77	Normal	ES
E12	2.12	1.84	Normal	ES
E13	2.09	1.84	Normal	ES
E14	2.05	1.64	Normal	ES
E15	2.12	1.98	Normal	ES
E16	2.13	1.77	Normal	ES
E17	2.14	2.08	Normal	ES
E18	2.17	2.05	Normal	ES
E19	2.09	1.81	Normal	ES
E20	2.07	1.53	Normal	ES
E21	2.11	1.88	Normal	ES
E22	2.11	1.87	Normal	ES
E23	2.13	0.79	Normal	ES
E24	0.33	0.26	Normal	ES
E25	2.06	1.68	Normal	ES
E26	2.18	0.5	Normal	ES
E27	2.38	0.63	Normal	ES
E28	1.24	0.76	Normal	ES
E29	1.11	1.73	Normal	ES
E30	1.42	0.32	Normal	ES
E31	1.71	0.49	Normal	ES
E32	1.33	0.63	Normal	ES
E33	1.96	0.81	Normal	ES
E34	2.11	2.06	Normal	ES
E35	2.03	0.45	Normal	ES
E36	1.65	1.06	Normal	ES
E37	1.75	1.33	Normal	ES
E38	2.08	1.34	Normal	ES
E39	2.01	0.98	Normal	ES
E40	1.93	0.71	Normal	ES
N1	2.03	2.05	Normal	N-ES
N2	2.12	1.66	Normal	N-ES
N3	2.26	2.19	Normal	N-ES

N4	2.1	1.72	Normal	N-ES
N5	2.08	1.93	Normal	N-ES
N6	2.08	1.85	Normal	N-ES
N7	1.98	1.28	Normal	N-ES
N8	2.11	1.48	Normal	N-ES
N9	2.04	1.51	Normal	N-ES
N10	2.15	1.86	Normal	N-ES
N11	1.97	1.52	Normal	N-ES
N12	2.11	1.92	Normal	N-ES
N13	2.2	1.96	Normal	N-ES
N14	2.11	2.05	Normal	N-ES
N15	2.13	1.93	Normal	N-ES
N16	2.11	1.69	Normal	N-ES
N17	2.14	1.73	Normal	N-ES
N18	2.06	1.4	Normal	N-ES
N19	2.02	1.45	Normal	N-ES
N20	2.1	1.06	Normal	N-ES
N21	1.87	0.71	Normal	N-ES
N22	2.15	1.24	Normal	N-ES
N23	2.11	1.28	Normal	N-ES
N24	1.85	1.46	Normal	N-ES
N25	2.09	1.17	Normal	N-ES
N26	1.25	0.61	Normal	N-ES
N27	2.14	0.5	Normal	N-ES
N28	2.27	1.08	Normal	N-ES
N29	1.63	0.51	Normal	N-ES
N30	1.31	0.42	Normal	N-ES
N31	1.72	1.09	Normal	N-ES
N32	2.1	1.76	Normal	N-ES
N33	2.15	2	Normal	N-ES
N34	2.08	2.13	Normal	N-ES
N35	2.07	1.93	Normal	N-ES
N36	1.84	1.18	Normal	N-ES
N37	1.84	1.24	Normal	N-ES
N38	2.05	1.59	Normal	N-ES
N39	1.95	0.93	Normal	N-ES
N40	1.77	0.58	Normal	N-ES

Table S3. The adaptor and barcode sequences used for PCR amplification.

Sample ID	Index 2	Index 1	Group
E1	CGTACGTA	GAAGTGTA	ES
E2	GAGTGGAT	GATCCTAT	ES
E3	ACTGATAT	GATCCTAT	ES
E4	ATTCCTTT	GATCCTAT	ES
E5	TATAGCCT	GATCCTAT	ES
E6	ATAGAGGC	GATCCTAT	ES
E7	CCTATCCT	GATCCTAT	ES
E8	GGCTCTGA	GATCCTAT	ES
E9	CGTACGTA	CGTACTAG	ES
E10	GAGTGGAT	CGTACTAG	ES
E11	ACTGATAT	GAAGTGTA	ES
E12	CCTATCCT	GAATTCGT	ES
E13	GGCTCTGA	GAATTCGT	ES
E14	CGTACGTA	GCACTTGC	ES
E15	GAGTGGAT	GCACTTGC	ES
E16	ACTGATAT	GCACTTGC	ES
E17	ATTCCTTT	GCACTTGC	ES
E18	ATTCCTTT	GAAGTGTA	ES
E19	TATAGCCT	GAAGTGTA	ES
E20	ATAGAGGC	GAAGTGTA	ES
E21	CCTATCCT	GAAGTGTA	ES
E22	CGTACGTA	GATCCTAT	ES
E23	CCTATCCT	GTGCCTGC	ES
E24	ATTCCTTT	GGCTAGAC	ES
E25	GGCTCTGA	GCACTTGC	ES
E26	TATAGCCT	CGGCTATG	ES
E27	ATAGAGGC	CGGCTATG	ES
E28	CCTATCCT	CGGCTATG	ES
E29	GGCTCTGA	CGGCTATG	ES
E30	CGTACGTA	TCCGCGAA	ES
E31	GAGTGGAT	TCCGCGAA	ES
E32	ATTCCTTT	TCCGCGAA	ES
E33	TATAGCCT	TCCGCGAA	ES
E34	ATAGAGGC	TCCGCGAA	ES
E35	CCTATCCT	TCCGCGAA	ES
E36	GGCTCTGA	TCCGCGAA	ES
E37	ATAGAGGC	GTGCCTGC	ES
E38	ATTCCTTT	CTGAAGCT	ES
E39	ATAGAGGC	CTGAAGCT	ES
E40	CCTATCCT	CTGAAGCT	ES
N1	GGCTCTGA	GTGCCTGC	N-ES
N2	GGCTCTGA	CGTACTAG	N-ES
N3	CGTACGTA	GAATTCGT	N-ES
N4	GAGTGGAT	GAATTCGT	N-ES
N5	ACTGATAT	GAATTCGT	N-ES
N6	ATTCCTTT	GAATTCGT	N-ES
N7	TATAGCCT	GAATTCGT	N-ES
N8	ATAGAGGC	GAATTCGT	N-ES
N9	TATAGCCT	GCACTTGC	N-ES

N10	GGCTCTGA	GAAGTGTA	N-ES
N11	GGCTCTGA	CTGAAGCT	N-ES
N12	GAGTGGAT	GAAGTGTA	N-ES
N13	CGTACGTA	GGCTAGAC	N-ES
N14	GAGTGGAT	GGCTAGAC	N-ES
N15	ACTGATAT	GGCTAGAC	N-ES
N16	TATAGCCT	GGCTAGAC	N-ES
N17	ATAGAGGC	GGCTAGAC	N-ES
N18	CCTATCCT	GGCTAGAC	N-ES
N19	GGCTCTGA	GGCTAGAC	N-ES
N20	CGTACGTA	CGGCTATG	N-ES
N21	GAGTGGAT	CGGCTATG	N-ES
N22	ACTGATAT	CGGCTATG	N-ES
N23	ACTGATAT	CGTACTAG	N-ES
N24	ATTCCTTT	CGGCTATG	N-ES
N25	ACTGATAT	TCCGCGAA	N-ES
N26	CGTACGTA	CTGAAGCT	N-ES
N27	CGTACGTA	GTGCCTGC	N-ES
N28	GAGTGGAT	GTGCCTGC	N-ES
N29	ACTGATAT	GTGCCTGC	N-ES
N30	ATTCCTTT	GTGCCTGC	N-ES
N31	TATAGCCT	GTGCCTGC	N-ES
N32	GAGTGGAT	CTGAAGCT	N-ES
N33	ACTGATAT	CTGAAGCT	N-ES
N34	ATTCCTTT	CGTACTAG	N-ES
N35	TATAGCCT	CTGAAGCT	N-ES
N36	TATAGCCT	CGTACTAG	N-ES
N37	ATAGAGGC	CGTACTAG	N-ES
N38	CCTATCCT	CGTACTAG	N-ES
N39	ATAGAGGC	GCACTTGC	N-ES
N40	CCTATCCT	GCACTTGC	N-ES

Table S4. The information of sequencing data and the α-diversity for each sample.

Sample ID	Reads number	Observed_ OTUs	Chao1	Simpson	Shannon	PD_whole	The Good' coverage	Group
E1	79824	738	772.4375	0.9392	5.74	48.3065	0.9991	ES
E2	79427	751	765.5	0.8575	5.7128	49.0497	0.9995	ES
E3	80352	767	775.7241	0.9878	8.0006	49.5206	0.9995	ES
E4	79857	754	764.5	0.9448	6.5778	48.578	0.9996	ES
E5	79799	739	777.9268	0.8971	5.6965	47.7737	0.999	ES
E6	80349	719	748.5	0.9251	5.4916	47.3048	0.999	ES
E7	79572	749	770.9688	0.9866	7.8234	48.4493	0.9991	ES
E8	79685	773	822.1364	0.9768	7.1726	49.7373	0.999	ES
E9	79650	768	782.625	0.9858	7.8608	49.0762	0.9994	ES
E10	79733	772	795.1562	0.9807	7.5436	49.6352	0.9992	ES
E11	79877	757	771.5312	0.9496	6.5211	49.2078	0.9995	ES
E12	79739	758	785.5122	0.9402	6.0736	48.8017	0.9992	ES
E13	79652	685	753.2208	0.8553	4.4875	46.1182	0.9985	ES
E14	79548	736	754.6364	0.9056	5.4568	47.8884	0.9994	ES
E15	79628	686	772.6234	0.9038	4.9671	45.3678	0.9983	ES
E16	79238	695	738.6974	0.9033	4.8921	45.92	0.9988	ES
E17	79916	743	774.4286	0.9152	5.4498	48.4431	0.9992	ES
E18	79808	756	791.15	0.9207	6.3664	49.4521	0.9993	ES
E19	80024	757	775.6	0.9394	6.7138	49.3362	0.9994	ES

E20	80116	703	781.7639	0.9011	5.3964	46.1982	0.9984	ES
E21	79825	719	757.2941	0.9048	5.4493	47.1377	0.999	ES
E22	79673	750	768	0.8802	5.3607	48.1999	0.9993	ES
E23	79482	712	754.5373	0.9169	5.3369	46.3529	0.9988	ES
E24	80225	694	746.4138	0.9031	5.1534	46.5349	0.9986	ES
E25	79735	723	751.6515	0.9122	5.3847	47.4915	0.999	ES
E26	79583	674	772.25	0.8859	4.92	45.5754	0.9981	ES
E27	80201	688	780.2625	0.9264	5.0116	45.5187	0.9983	ES
E28	79420	760	773.5227	0.9643	7.1031	49.0817	0.9994	ES
E29	79531	721	747.6667	0.8826	5.144	46.6418	0.9991	ES
E30	79551	732	817.1538	0.9214	5.258	47.5041	0.9988	ES
E31	79521	701	740	0.8646	4.8936	47.163	0.9989	ES
E32	79535	671	714.0112	0.9478	5.6318	45.0829	0.9987	ES
E33	79787	717	761.069	0.8803	4.9131	47.9843	0.9989	ES
E34	79624	726	750.2967	0.8978	5.1052	48.0228	0.999	ES
E35	79851	711	791.2941	0.8909	4.6811	47.2993	0.9985	ES
E36	80118	729	786.2143	0.926	5.3517	47.6303	0.9987	ES
E37	79642	662	721.4125	0.8669	4.542	44.5274	0.9986	ES
E38	79984	711	758.7885	0.9579	6.418	46.6574	0.9988	ES
E39	79535	744	775.6389	0.7863	4.622	48.626	0.999	ES
E40	79683	716	764.6143	0.7095	3.8643	47.1408	0.9986	ES

N1	79517	265	351.8889	0.9297	4.8129	21.9524	0.999	N-ES
N2	79535	707	769.4776	0.8758	4.9549	45.0546	0.9986	N-ES
N3	79744	546	640.25	0.9045	5.1679	36.471	0.9983	N-ES
N4	80041	630	736.4189	0.9158	5.0971	41.0811	0.9982	N-ES
N5	79843	581	674.6133	0.9145	4.6914	39.2518	0.9984	N-ES
N6	79928	614	713.1852	0.8694	4.7159	40.3971	0.9985	N-ES
N7	79873	565	696.2917	0.9121	4.8133	38.767	0.9981	N-ES
N8	79670	335	392	0.9187	4.4178	26.5081	0.9986	N-ES
N9	79559	446	517.3659	0.9353	5.1551	34.1919	0.9989	N-ES
N10	79734	538	729.3438	0.8579	4.1139	35.9751	0.9978	N-ES
N11	79426	551	624.1231	0.9382	5.1309	36.659	0.9986	N-ES
N12	79809	543	628.5932	0.8877	4.7473	37.2703	0.9986	N-ES
N13	79698	591	657.1235	0.9069	4.7663	39.3369	0.9986	N-ES
N14	79369	572	638	0.8929	4.5586	38.0055	0.9986	N-ES
N15	79042	523	598.039	0.8123	4.0413	35.7982	0.9985	N-ES
N16	79761	559	658.122	0.9045	4.4724	37.3441	0.9982	N-ES
N17	79575	538	667.4932	0.8559	4.6145	38.4346	0.998	N-ES
N18	79772	672	732.1646	0.9223	4.9679	42.3644	0.9986	N-ES
N19	80059	612	705.7875	0.9176	5.1895	40.4939	0.9983	N-ES
N20	79829	617	709.2625	0.8582	4.3552	39.8581	0.9983	N-ES
N21	79715	641	726.8987	0.8871	4.8637	41.6694	0.9983	N-ES

N22	79494	655	744.3187	0.8514	4.2784	44.8611	0.9982	N-ES
N23	79847	564	648	0.8602	4.4599	37.4275	0.9983	N-ES
N24	79857	579	691.241	0.9244	5.0858	38.6684	0.9981	N-ES
N25	79993	605	698	0.9138	4.876	42.8274	0.9983	N-ES
N26	80199	588	683.2841	0.9389	5.4883	38.5641	0.9982	N-ES
N27	79461	570	693.3448	0.9178	4.6258	38.484	0.9979	N-ES
N28	79887	596	677.5789	0.9242	5.2617	41.666	0.9982	N-ES
N29	80056	595	699.6742	0.8445	4.2371	41.4879	0.9981	N-ES
N30	79779	655	774.6582	0.8581	4.6639	44.3582	0.9981	N-ES
N31	79722	651	733.5	0.8707	4.5441	41.9923	0.9983	N-ES
N32	79553	664	728.7093	0.8566	5.0201	42.3529	0.9985	N-ES
N33	79782	543	676.0435	0.8967	4.5519	36.8822	0.998	N-ES
N34	79559	492	615.0469	0.9022	4.5387	33.116	0.9981	N-ES
N35	79477	514	643.1449	0.8371	3.9907	37.0208	0.998	N-ES
N36	80401	505	638.0154	0.9087	4.6374	36.7247	0.9981	N-ES
N37	79780	563	614.3025	0.9091	4.9237	39.7047	0.9984	N-ES
N38	79558	631	703.225	0.8673	4.786	40.8077	0.9985	N-ES
N39	79576	611	689.9789	0.892	4.5216	42.5813	0.9983	N-ES
N40	79558	596	685.7391	0.8627	4.4245	41.9657	0.9982	N-ES

Table S5. Differential bacteria genus between ES and N-ES groups.

Phylum	Genus	ES		N-ES		Fold change (ES/N-ES)	Enrichment	P value
		Mean	SD	Mean	SD			
Proteobacteria	uncultured_f_Xanthobacteraceae	1.90E-03	2.17E-03	1.33E-04	7.61E-05	14.30	ES	2.09E-17
Firmicutes	Weissella	2.12E-04	1.42E-04	2.47E-03	2.49E-03	0.09	N-ES	4.38E-16
Cyanobacteria	Phaseolus_acutifolius_tepary_bean	3.31E-04	2.28E-04	4.11E-03	4.39E-03	0.08	N-ES	8.20E-15
Proteobacteria	Bradyrhizobium	1.25E-04	9.76E-05	7.63E-04	6.81E-04	0.16	N-ES	3.33E-14
Proteobacteria	Pseudomonas	5.22E-04	2.28E-04	3.18E-03	3.77E-03	0.16	N-ES	6.32E-14
Actinobacteria	Cutibacterium	1.07E-04	8.23E-05	6.41E-04	5.76E-04	0.17	N-ES	6.97E-14
Acidobacteria	Bryobacter	1.40E-03	1.78E-03	5.91E-05	4.89E-05	23.60	ES	2.01E-12
Acidobacteria	RB41	2.75E-03	2.99E-03	1.20E-04	1.41E-04	22.83	ES	2.36E-12
Proteobacteria	Neisseria	3.43E-05	3.37E-05	7.18E-04	7.48E-04	0.05	N-ES	2.71E-12
Acidobacteria	uncultured_o_11-24	1.07E-05	1.74E-05	5.46E-04	7.06E-04	0.02	N-ES	4.13E-12
Proteobacteria	uncultured_f_TRA3-20	6.39E-05	5.68E-05	1.42E-03	1.71E-03	0.05	N-ES	4.13E-12
Actinobacteria	Streptomyces	1.30E-03	1.72E-03	4.74E-05	5.19E-05	27.38	ES	4.13E-12
Cyanobacteria	uncultured_o_Chloroplast	3.86E-03	5.51E-03	3.66E-04	2.98E-04	10.55	ES	4.14E-12
Fusobacteria	Cetobacterium	1.05E-03	1.21E-03	7.27E-05	6.57E-05	14.40	ES	4.32E-12
Proteobacteria	uncultured_f_Reyranellaceae	1.81E-05	2.19E-05	4.59E-04	5.68E-04	0.04	N-ES	4.57E-12
Proteobacteria	uncultured_c_Alphaproteobacteria	1.26E-03	1.96E-03	4.56E-05	4.17E-05	27.67	ES	4.57E-12
Firmicutes	Coprococcus_1	1.11E-03	1.26E-03	7.55E-05	7.01E-05	14.72	ES	4.57E-12
Rokubacteria	uncultured_o_Rokubacteriales	1.32E-03	1.56E-03	7.83E-05	6.22E-05	16.82	ES	5.39E-12
Gemmatimonadetes	uncultured_f_Longimicrobiaceae	7.09E-06	1.25E-05	3.61E-04	4.68E-04	0.02	N-ES	5.39E-12
Proteobacteria	uncultured_f_KF-JG30-B3	4.97E-04	6.05E-04	2.90E-05	2.64E-05	17.16	ES	5.39E-12
Acidobacteria	Stenotrophobacter	9.99E-06	1.26E-05	2.48E-04	2.80E-04	0.04	N-ES	5.39E-12
Proteobacteria	Dongia	1.96E-03	2.39E-03	1.10E-04	9.02E-05	17.80	ES	5.39E-12
Actinobacteria	Brevibacterium	2.40E-04	3.67E-04	1.68E-03	1.97E-03	0.14	N-ES	5.39E-12
Gemmatimonadetes	uncultured_f_Gemmatimonadaceae	1.37E-03	1.67E-03	9.15E-05	7.20E-05	14.98	ES	5.68E-12
Proteobacteria	uncultured_c_Deltaproteobacteria	1.16E-03	1.18E-03	8.55E-05	8.47E-05	13.54	ES	5.68E-12
Firmicutes	Staphylococcus	1.23E-03	1.29E-03	1.00E-04	1.04E-04	12.25	ES	5.68E-12
Proteobacteria	MND1	1.61E-03	1.95E-03	7.49E-05	6.01E-05	21.52	ES	5.68E-12
Acidobacteria	uncultured_o_uncultivated_soil_clone_C112	4.57E-04	4.86E-04	2.63E-05	2.99E-05	17.37	ES	6.21E-12

Actinobacteria	uncultured_o_Microtrichales	3.34E-05	4.05E-05	9.99E-04	1.28E-03	0.03	N-ES	6.21E-12
Acidobacteria	uncultured_f_Blastocatellaceae	8.89E-04	9.15E-04	6.82E-05	4.62E-05	13.04	ES	7.02E-12
Actinobacteria	uncultured_o_Gaiellales	5.02E-04	6.76E-04	2.04E-05	2.82E-05	24.61	ES	7.84E-12
Chloroflexi	uncultured_c_KD4-96	5.21E-04	6.30E-04	2.86E-05	2.69E-05	18.20	ES	8.28E-12
Proteobacteria	Pedomicrobium	4.87E-04	6.07E-04	2.30E-05	1.99E-05	21.20	ES	8.93E-12
Epsilonbacteraeota	Sulfurovum	2.86E-04	3.00E-04	1.80E-05	2.10E-05	15.88	ES	9.51E-12
Firmicutes	Leuconostoc	2.65E-03	2.78E-03	1.71E-04	1.45E-04	15.53	ES	9.51E-12
Actinobacteria	uncultured_p_Actinobacteria	3.19E-04	3.54E-04	1.40E-05	2.20E-05	22.76	ES	1.10E-11
Nitrospirae	Nitrospira	7.27E-04	7.66E-04	4.96E-05	5.54E-05	14.67	ES	1.16E-11
Firmicutes	Lactococcus	9.86E-04	1.29E-03	5.84E-05	6.79E-05	16.90	ES	1.16E-11
Firmicutes	Ruminococcaceae_UCG-014	5.99E-03	7.03E-03	7.54E-04	1.11E-03	7.95	ES	1.18E-11
Proteobacteria	Steroidobacter	3.41E-05	3.32E-05	6.04E-04	6.90E-04	0.06	N-ES	1.24E-11
Actinobacteria	Pseudonocardia	2.24E-05	3.41E-05	6.57E-04	9.05E-04	0.03	N-ES	1.24E-11
Actinobacteria	Virgisporangium	1.32E-05	1.84E-05	2.52E-04	3.16E-04	0.05	N-ES	1.27E-11
Proteobacteria	Ochrobactrum	1.10E-04	9.63E-05	6.64E-04	6.10E-04	0.17	N-ES	1.35E-11
Actinobacteria	Solirubrobacter	1.02E-03	1.38E-03	4.17E-05	4.25E-05	24.54	ES	1.36E-11
Proteobacteria	Reyranella	1.04E-04	6.83E-05	1.04E-03	1.17E-03	0.10	N-ES	1.38E-11
Actinobacteria	Corynebacterium_1	9.26E-04	1.20E-03	8.87E-05	9.28E-05	10.44	ES	1.45E-11
Proteobacteria	uncultured_f_Geminicoccaceae	3.04E-04	3.75E-04	1.30E-05	2.34E-05	23.50	ES	1.66E-11
Actinobacteria	Longispora	1.04E-03	1.41E-03	3.56E-05	4.53E-05	29.37	ES	2.88E-11
Firmicutes	Peptostreptococcus	4.07E-04	5.86E-04	2.44E-05	2.42E-05	16.69	ES	2.88E-11
Actinobacteria	uncultured_o_IMCC26256	1.85E-04	2.19E-04	8.25E-06	1.73E-05	22.39	ES	3.91E-11
Proteobacteria	uncultured_f_Burkholderiaceae	4.72E-04	4.69E-04	4.97E-05	5.16E-05	9.51	ES	4.01E-11
Proteobacteria	uncultured_f_Mitochondria	1.72E-04	1.98E-04	1.50E-03	1.73E-03	0.11	N-ES	4.27E-11
Proteobacteria	Ralstonia	2.01E-04	2.17E-04	1.80E-05	2.33E-05	11.15	ES	4.27E-11
Chloroflexi	uncultured_f_AKYG1722	1.92E-04	3.01E-04	4.99E-06	9.99E-06	38.42	ES	4.71E-11
Actinobacteria	uncultured_f_Pseudonocardiaceae	4.77E-04	6.53E-04	1.46E-05	2.28E-05	32.72	ES	4.84E-11
Proteobacteria	Brevundimonas	7.68E-05	6.62E-05	6.17E-04	6.74E-04	0.12	N-ES	5.30E-11
Verrucomicrobia	Haloferula	1.53E-04	1.76E-04	9.91E-06	1.76E-05	15.44	ES	5.30E-11
Bacteroidetes	uncultured_f_Microscillaceae	3.25E-04	3.84E-04	1.56E-05	2.28E-05	20.88	ES	5.52E-11
Proteobacteria	Rhodoplanes	1.87E-04	2.29E-04	1.02E-05	1.74E-05	18.35	ES	5.98E-11
Actinobacteria	Actinophytocola	4.46E-04	6.30E-04	1.20E-05	2.04E-05	37.09	ES	5.98E-11

Actinobacteria	Mycobacterium	3.42E-04	4.59E-04	2.10E-05	2.77E-05	16.28	ES	7.57E-11
Actinobacteria	uncultured_f_67-14	1.08E-03	1.34E-03	7.05E-05	6.57E-05	15.26	ES	8.16E-11
Acidobacteria	Subgroup_10	2.69E-04	3.33E-04	1.02E-05	1.53E-05	26.29	ES	8.60E-11
Proteobacteria	Rahnella	8.36E-04	8.59E-04	5.49E-05	1.10E-04	15.23	ES	8.60E-11
Firmicutes	Anaerovibrio	1.81E-04	2.11E-04	1.11E-05	2.38E-05	16.32	ES	8.60E-11
Proteobacteria	Sphingomonas	2.24E-04	1.76E-04	1.51E-03	1.35E-03	0.15	N-ES	8.76E-11
Gemmatimonadetes	uncultured_c_S0134_terrestrial_group	2.98E-04	3.88E-04	7.82E-06	1.29E-05	38.10	ES	8.85E-11
Actinobacteria	Dactylosporangium	1.66E-04	2.18E-04	5.29E-06	8.22E-06	31.40	ES	9.00E-11
Proteobacteria	Shewanella	4.97E-04	5.76E-04	3.87E-05	3.91E-05	12.84	ES	9.93E-11
Verrucomicrobia	Candidatus_Udaeobacter	2.97E-04	3.26E-04	1.67E-05	2.20E-05	17.86	ES	1.06E-10
Proteobacteria	uncultured_o_CCD24	4.62E-04	5.36E-04	3.83E-05	3.58E-05	12.07	ES	1.16E-10
Proteobacteria	Ellin6067	1.92E-04	2.12E-04	1.21E-05	1.65E-05	15.93	ES	1.24E-10
Acidobacteria	uncultured_o_Subgroup_7	4.16E-05	3.27E-05	6.19E-04	7.57E-04	0.07	N-ES	1.29E-10
Acidobacteria	uncultured_o_Acidobacteria_IGE-010	6.01E-06	1.35E-05	1.95E-04	2.72E-04	0.03	N-ES	1.29E-10
Acidobacteria	Candidatus_Solibacter	7.79E-04	8.91E-04	8.03E-05	5.26E-05	9.71	ES	1.48E-10
Actinobacteria	Actinomadura	2.69E-04	3.84E-04	6.39E-06	1.16E-05	42.07	ES	1.63E-10
Proteobacteria	Phenylobacterium	2.29E-04	2.45E-04	2.51E-05	2.25E-05	9.15	ES	1.76E-10
Actinobacteria	Kibdelosporangium	1.84E-04	2.49E-04	8.21E-06	1.21E-05	22.42	ES	1.81E-10
Proteobacteria	Rhodomicrobium	4.06E-04	5.32E-04	1.53E-05	2.04E-05	26.53	ES	1.84E-10
Proteobacteria	uncultured_f_Desulfarculaceae	1.40E-04	1.82E-04	6.41E-06	1.30E-05	21.88	ES	2.53E-10
Acidobacteria	uncultured_o_Acidobacteriales	7.26E-04	6.28E-04	1.15E-04	1.03E-04	6.29	ES	2.80E-10
Atribacteria	uncultured_c_JS1	3.81E-04	4.82E-04	3.37E-05	3.12E-05	11.31	ES	2.82E-10
Planctomycetes	AKYG587	1.67E-04	2.41E-04	5.62E-06	1.09E-05	29.71	ES	2.83E-10
Proteobacteria	Variovorax	1.69E-04	1.68E-04	2.21E-05	2.67E-05	7.66	ES	3.54E-10
Bacteroidetes	Prevotella_7	5.86E-04	2.38E-03	2.96E-05	3.01E-05	19.77	ES	3.54E-10
Firmicutes	Gemella	1.85E-04	2.16E-04	2.93E-05	2.99E-05	6.29	ES	3.54E-10
Proteobacteria	uncultured_o_Gammaproteobacteria_Incertae_Sedis	7.54E-04	7.90E-04	1.32E-04	9.57E-05	5.73	ES	3.60E-10
Chloroflexi	uncultured_c_TK10	2.76E-04	3.49E-04	1.18E-05	2.02E-05	23.39	ES	5.20E-10
Proteobacteria	Tepidimonas	1.86E-04	2.15E-04	9.38E-05	4.78E-04	1.98	ES	5.20E-10
Actinobacteria	Kribbella	2.20E-04	3.07E-04	1.08E-05	1.90E-05	20.42	ES	5.26E-10
Proteobacteria	SWB02	2.69E-04	3.61E-04	1.70E-05	2.32E-05	15.82	ES	5.70E-10

Bacteroidetes	Sungkyunkwania	5.40E-04	1.74E-03	1.78E-05	2.00E-05	30.38	ES	5.91E-10
Bacteroidetes	Prevotellaceae_UCG-001	6.92E-04	1.84E-03	1.89E-04	1.06E-03	3.66	ES	6.25E-10
Bacteroidetes	Prevotella_9	9.94E-03	1.14E-02	1.72E-03	5.69E-04	5.78	ES	6.25E-10
Actinobacteria	Iamia	2.70E-04	3.31E-04	1.38E-05	1.71E-05	19.60	ES	6.57E-10
Acidobacteria	uncultured_o_Acidobacteria_IGE-011	4.88E-05	3.86E-05	3.89E-04	4.21E-04	0.13	N-ES	7.24E-10
Proteobacteria	uncultured_f_Sandaracinaceae	3.25E-04	4.33E-04	1.03E-05	2.10E-05	31.70	ES	7.33E-10
Actinobacteria	uncultured_c_MB-A2-108	1.74E-04	2.25E-04	5.67E-06	1.05E-05	30.61	ES	7.56E-10
Firmicutes	Psychrobacillus	1.53E-04	1.89E-04	6.09E-06	9.62E-06	25.07	ES	8.42E-10
Proteobacteria	Haliangium	4.06E-04	5.10E-04	3.28E-05	2.88E-05	12.38	ES	8.90E-10
Actinobacteria	Flindersiella	2.74E-04	3.81E-04	8.20E-06	1.21E-05	33.38	ES	1.00E-09
Proteobacteria	uncultured_o_Rhizobiales	3.01E-04	4.06E-04	1.71E-05	2.12E-05	17.64	ES	1.10E-09
Actinobacteria	Dietzia	2.98E-04	3.47E-04	5.33E-05	1.12E-04	5.58	ES	1.15E-09
Proteobacteria	Serratia	2.44E-04	1.61E-04	1.18E-03	1.08E-03	0.21	N-ES	1.15E-09
Chloroflexi	uncultured_c_Gitt-GS-136	4.91E-04	7.14E-04	2.21E-05	2.18E-05	22.21	ES	1.20E-09
Actinobacteria	Rothia	1.81E-04	2.44E-04	1.22E-03	1.34E-03	0.15	N-ES	1.39E-09
Proteobacteria	Hirschia	2.01E-04	2.65E-04	8.13E-06	1.19E-05	24.67	ES	1.39E-09
Bacteroidetes	Terrimonas	1.46E-04	1.50E-04	1.72E-05	2.50E-05	8.47	ES	1.41E-09
Actinobacteria	uncultured_o_Actinomarinales	2.33E-04	3.08E-04	1.27E-05	2.07E-05	18.29	ES	1.45E-09
Proteobacteria	uncultured_f_Sphingomonadaceae	5.33E-04	7.37E-04	2.90E-05	4.15E-05	18.35	ES	1.52E-09
Actinobacteria	Rhodococcus	2.01E-04	2.08E-04	2.74E-05	6.54E-05	7.33	ES	1.56E-09
Proteobacteria	uncultured_f_Desulfovibrionaceae	2.05E-04	1.71E-04	9.31E-04	7.88E-04	0.22	N-ES	2.18E-09
Verrucomicrobia	Akkermansia	4.90E-03	3.54E-03	1.85E-03	3.50E-03	2.65	ES	2.18E-09
Proteobacteria	Herbaspirillum	1.61E-04	1.29E-04	4.33E-05	3.34E-05	3.73	ES	3.59E-09
Bacteroidetes	Prevotella_1	1.52E-04	1.71E-04	1.33E-05	1.75E-05	11.46	ES	4.30E-09
Proteobacteria	Shimia	1.46E-04	3.06E-04	6.73E-06	1.55E-05	21.69	ES	4.82E-09
Firmicutes	Enterococcus	8.24E-04	9.48E-04	1.12E-04	2.05E-04	7.34	ES	4.87E-09
Proteobacteria	Delftia	1.91E-04	1.78E-04	3.59E-05	2.81E-05	5.33	ES	4.87E-09
Proteobacteria	Hydrogenophaga	1.82E-04	1.88E-04	3.99E-05	1.09E-04	4.57	ES	4.95E-09
Verrucomicrobia	Opitutus	1.54E-04	1.87E-04	1.27E-05	1.69E-05	12.19	ES	5.23E-09
Acidobacteria	uncultured_f_Acidobacteriaceae_Subgroup_1	2.46E-04	2.72E-04	3.28E-05	3.01E-05	7.50	ES	5.92E-09
Verrucomicrobia	Luteolibacter	1.46E-04	1.84E-04	7.10E-06	1.54E-05	20.63	ES	6.15E-09

Bacteroidetes	Prevotellaceae_UCG-003	8.74E-04	1.13E-03	8.03E-05	1.74E-04	10.88	ES	8.17E-09
Proteobacteria	Acinetobacter	2.52E-04	3.37E-04	1.14E-03	1.10E-03	0.22	N-ES	9.02E-09
Proteobacteria	uncultured_o_PLTA13	2.64E-04	3.19E-04	2.70E-05	2.42E-05	9.78	ES	1.13E-08
Bacteroidetes	Cloacibacterium	1.30E-04	1.30E-04	1.23E-04	6.70E-04	1.06	ES	1.28E-08
Actinobacteria	Microbacterium	1.92E-04	1.74E-04	3.50E-05	4.67E-05	5.48	ES	1.33E-08
Bacteroidetes	uncultured_f_Flavobacteriaceae	3.16E-04	7.44E-04	2.10E-05	2.67E-05	15.03	ES	1.70E-08
Bacteroidetes	Prevotellaceae_UCG-004	1.27E-04	1.81E-04	8.08E-06	3.28E-05	15.66	ES	1.84E-08
Actinobacteria	Micrococcus	1.70E-04	1.98E-04	2.58E-05	4.55E-05	6.56	ES	1.95E-08
Proteobacteria	Succinivibrio	1.06E-03	1.66E-03	6.74E-05	2.44E-04	15.67	ES	2.19E-08
Proteobacteria	uncultured_f_Rhizobiaceae	1.35E-04	1.71E-04	1.78E-05	1.81E-05	7.57	ES	2.78E-08
Firmicutes	Proteiniclasticum	1.99E-04	2.59E-04	1.74E-05	2.59E-05	11.45	ES	3.04E-08
Proteobacteria	agricultural_soil_bacterium_SC-I-84	1.69E-04	1.82E-04	1.76E-05	2.49E-05	9.60	ES	3.44E-08
Proteobacteria	Dokdonella	1.62E-04	1.41E-04	4.17E-05	5.24E-05	3.88	ES	1.11E-07
Proteobacteria	Devosia	3.72E-04	4.24E-04	7.60E-05	7.01E-05	4.90	ES	1.13E-07
Epsilonbacteraeota	Arcobacter	1.95E-04	5.95E-04	1.75E-05	2.62E-05	11.14	ES	1.70E-07
Firmicutes	Lactobacillus	1.85E-02	1.94E-02	9.47E-03	2.56E-02	1.95	ES	1.93E-07
Proteobacteria	Nordella	2.38E-04	2.81E-04	2.18E-05	1.77E-05	10.93	ES	2.39E-07
Proteobacteria	Pseudolabrys	1.09E-04	1.08E-04	2.30E-05	2.86E-05	4.75	ES	2.47E-07
Actinobacteria	Nonomuraea	1.98E-04	2.63E-04	6.77E-06	1.08E-05	29.19	ES	2.47E-07
Firmicutes	uncultured_o_Lactobacillales	3.11E-04	3.27E-04	6.41E-05	9.91E-05	4.85	ES	2.53E-07
Proteobacteria	Motilimonas	1.27E-04	1.41E-04	1.14E-05	1.35E-05	11.20	ES	2.56E-07
Firmicutes	Pediococcus	4.00E-04	2.72E-04	1.53E-04	9.90E-05	2.62	ES	2.57E-07
Proteobacteria	uncultured_f_Steroidobacteraceae	1.51E-04	1.36E-04	2.76E-05	3.07E-05	5.48	ES	2.80E-07
Fusobacteria	Fusobacterium	4.69E-03	7.00E-03	1.83E-03	5.29E-03	2.56	ES	3.20E-07
Proteobacteria	Paracoccus	1.26E-04	1.17E-04	2.59E-05	3.89E-05	4.86	ES	3.32E-07
Cyanobacteria	Synechococcus_CC9902	1.52E-03	5.99E-03	2.92E-05	6.46E-05	52.03	ES	3.38E-07
Bacteroidetes	uncultured_f_Prevotellaceae	7.45E-04	9.41E-04	2.65E-04	5.41E-04	2.81	ES	3.78E-07
Bacteroidetes	Proteiniphilum	1.67E-04	3.21E-04	2.46E-05	4.92E-05	6.78	ES	3.79E-07
Proteobacteria	Hafnia-Obesumbacterium	1.05E-04	1.87E-04	7.06E-04	7.54E-04	0.15	N-ES	4.98E-07
Actinobacteria	Lawsonella	1.48E-04	1.76E-04	1.97E-05	2.63E-05	7.49	ES	4.98E-07
Firmicutes	Ruminococcaceae_UCG-005	1.17E-02	8.98E-03	4.40E-03	5.39E-03	2.65	ES	5.32E-07
Firmicutes	Dubosiella	7.05E-04	5.74E-04	2.08E-04	2.28E-04	3.40	ES	7.47E-07

Bacteroidetes	Alloprevotella	4.20E-03	1.30E-02	4.79E-03	1.76E-02	0.88	N-ES	8.00E-07
Proteobacteria	Allorhizobium-Neorhizobium-Pararhizobium-Rhizobium	5.95E-04	4.59E-04	2.03E-04	1.52E-04	2.93	ES	8.95E-07
Bacteroidetes	Chryseolinea	1.38E-04	1.35E-04	3.11E-05	2.92E-05	4.43	ES	1.22E-06
Bacteroidetes	Rikenellaceae_RC9_gut_group	1.38E-03	1.31E-03	4.06E-04	2.89E-04	3.41	ES	2.10E-06
Proteobacteria	uncultured_f_Caulobacteraceae	1.29E-04	1.69E-04	2.16E-05	2.48E-05	5.98	ES	2.29E-06
Firmicutes	Lachnospiraceae_UCG-006	2.29E-04	2.26E-04	5.48E-05	6.82E-05	4.18	ES	2.48E-06
Patescibacteria	Candidatus_Saccharimonas	4.83E-04	5.24E-04	9.38E-05	1.31E-04	5.15	ES	2.84E-06
Spirochaetes	uncultured_f_Spirochaetaceae	1.43E-04	2.50E-04	1.31E-05	3.58E-05	10.93	ES	3.31E-06
Actinobacteria	Brachybacterium	3.66E-04	4.35E-04	1.02E-04	1.78E-04	3.60	ES	3.86E-06
Proteobacteria	Actinobacillus	9.39E-05	1.23E-04	4.67E-05	1.24E-04	2.01	ES	4.01E-06
Proteobacteria	Halocynthiibacter	1.52E-04	2.37E-04	1.55E-05	1.96E-05	9.82	ES	4.39E-06
Proteobacteria	Cronobacter	2.79E-04	5.14E-04	8.05E-05	2.41E-04	3.47	ES	4.63E-06
Firmicutes	[Eubacterium]_xylanophilum_group	3.02E-04	7.08E-04	1.98E-04	8.76E-04	1.53	ES	5.02E-06
Cyanobacteria	Pardosa_pseudoannulata	2.21E-04	8.40E-04	1.05E-05	1.78E-05	21.17	ES	5.63E-06
Proteobacteria	Cellvibrio	9.20E-05	8.11E-05	2.58E-04	1.46E-03	0.36	N-ES	6.05E-06
Firmicutes	Bacillus	4.40E-04	3.36E-04	1.66E-04	1.30E-04	2.65	ES	8.37E-06
Proteobacteria	Piscinibacter	2.13E-04	2.89E-04	2.83E-05	6.30E-05	7.53	ES	9.87E-06
Actinobacteria	uncultured_f_Microbacteriaceae	2.99E-04	2.47E-04	1.69E-04	4.99E-04	1.77	ES	1.28E-05
Proteobacteria	Bosea	1.54E-04	1.33E-04	4.72E-05	4.80E-05	3.27	ES	1.81E-05
Firmicutes	Sporanaerobacter	2.67E-04	4.71E-04	5.66E-05	6.58E-05	4.71	ES	1.83E-05
Bacteroidetes	Prevotellaceae_NK3B31_group	7.32E-04	3.05E-03	5.33E-05	4.70E-05	13.73	ES	2.04E-05
Proteobacteria	Acidovorax	1.82E-04	1.99E-04	3.95E-05	3.99E-05	4.61	ES	3.26E-05
Proteobacteria	uncultured_f_Enterobacteriaceae	6.18E-04	5.25E-04	4.24E-04	8.30E-04	1.46	ES	3.63E-05
Firmicutes	Candidatus_Arthromitus	1.71E-04	1.97E-04	4.59E-05	4.31E-05	3.72	ES	4.06E-05
Firmicutes	Lachnospiraceae_NK4A136_group	2.59E-03	4.04E-03	1.07E-03	1.22E-03	2.41	ES	7.26E-05
Epsilonbacteraeota	Helicobacter	5.86E-04	4.58E-04	1.38E-03	1.01E-03	0.42	N-ES	8.28E-05
Proteobacteria	Plesiomonas	5.89E-05	7.13E-05	8.36E-05	3.51E-04	0.70	N-ES	8.54E-05
Proteobacteria	Comamonas	3.53E-04	4.98E-04	2.90E-04	6.88E-04	1.22	ES	1.07E-04
Bacteroidetes	uncultured_o_Bacteroidales	8.95E-04	6.11E-04	5.07E-04	6.16E-04	1.77	ES	1.33E-04
Bacteroidetes	Bacteroides	8.02E-02	7.54E-02	1.63E-01	1.03E-01	0.49	N-ES	1.88E-04
Proteobacteria	Stenotrophomonas	1.47E-04	1.49E-04	5.23E-05	1.06E-04	2.81	ES	2.29E-04

Actinobacteria	uncultured_f_Micromonosporaceae	3.02E-04	3.02E-04	8.38E-05	7.07E-05	3.60	ES	2.51E-04
Acidobacteria	uncultured_c_Subgroup_17	1.26E-04	1.02E-04	5.30E-05	5.35E-05	2.37	ES	2.95E-04
Firmicutes	Lachnospiraceae_XPB1014_group	1.01E-04	6.71E-05	5.21E-05	3.41E-05	1.95	ES	3.26E-04
Proteobacteria	Thiobacillus	9.65E-05	1.00E-04	3.41E-05	3.42E-05	2.83	ES	3.32E-04
Bacteroidetes	uncultured_f_p-2534-18B5_gut_group	1.56E-04	2.59E-04	8.76E-06	3.58E-05	17.76	ES	4.10E-04
Firmicutes	Erysipelotrichaceae_UCG-003	1.02E-03	1.16E-03	2.37E-03	2.34E-03	0.43	N-ES	5.30E-04
Proteobacteria	Dechloromonas	9.38E-05	7.82E-05	4.14E-05	4.82E-05	2.26	ES	6.35E-04
Proteobacteria	Mesorhizobium	1.57E-04	1.30E-04	7.87E-05	4.86E-05	1.99	ES	8.78E-04
Firmicutes	uncultured_f_Ruminococcaceae	1.79E-03	5.82E-04	1.55E-03	1.65E-03	1.16	ES	9.35E-04
Actinobacteria	Eggerthella	1.04E-04	1.17E-04	4.93E-05	8.46E-05	2.12	ES	9.52E-04
Bacteroidetes	Rikenella	3.07E-04	2.20E-04	1.59E-04	1.20E-04	1.93	ES	1.23E-03
Firmicutes	Family_XIII_AD3011_group	1.20E-04	1.12E-04	7.08E-05	1.37E-04	1.70	ES	1.24E-03
Firmicutes	Lachnospiraceae_UCG-003	4.28E-04	2.37E-03	1.38E-04	7.55E-04	3.10	ES	1.47E-03
Deferribacteres	Mucispirillum	3.13E-04	3.09E-04	9.84E-05	6.92E-05	3.18	ES	1.65E-03
Proteobacteria	Desulfovibrio	3.04E-03	2.80E-03	1.95E-03	2.33E-03	1.56	ES	2.60E-03
Firmicutes	Intestinibacter	1.06E-03	1.69E-03	7.21E-04	1.44E-03	1.47	ES	2.97E-03
Proteobacteria	Gluconobacter	5.19E-04	3.70E-04	2.61E-04	1.96E-04	1.98	ES	2.97E-03
Firmicutes	Veillonella	3.08E-03	2.96E-03	2.61E-03	7.81E-03	1.18	ES	3.37E-03
Chloroflexi	uncultured_f_A4b	1.77E-04	1.13E-04	1.04E-04	9.65E-05	1.71	ES	4.15E-03
Firmicutes	Megasphaera	2.09E-02	2.86E-02	3.69E-02	4.46E-02	0.57	N-ES	4.97E-03
Bacteroidetes	uncultured_f_Muribaculaceae	4.69E-03	3.64E-03	3.29E-03	5.34E-03	1.43	ES	6.20E-03
Proteobacteria	Mailhella	4.12E-05	5.03E-05	1.16E-04	5.01E-04	0.35	N-ES	6.20E-03
Firmicutes	Ileibacterium	3.21E-04	5.10E-04	6.89E-04	8.36E-04	0.47	N-ES	7.58E-03
Actinobacteria	Coriobacteriaceae_UCG-002	2.11E-04	1.35E-04	1.44E-04	1.49E-04	1.47	ES	8.50E-03
Proteobacteria	Pantoea	3.44E-03	5.47E-03	2.80E-03	4.93E-03	1.23	ES	8.57E-03
Bacteroidetes	Sphingobacterium	1.06E-04	5.79E-05	7.41E-05	4.56E-05	1.43	ES	9.61E-03
Bacteroidetes	uncultured_f_Saprospiraceae	1.49E-04	6.14E-04	2.46E-06	7.68E-06	60.80	ES	9.81E-03
Firmicutes	uncultured_f_Christensenellaceae	1.81E-04	4.68E-04	7.06E-05	1.30E-04	2.56	ES	1.08E-02
Firmicutes	Erysipelatoclostridium	4.93E-04	5.84E-04	2.15E-04	2.71E-04	2.29	ES	1.13E-02
Firmicutes	Ruminiclostridium	4.80E-04	2.86E-04	3.16E-04	2.11E-04	1.52	ES	1.60E-02
Patescibacteria	uncultured_f_Saccharimonadaceae	8.78E-05	1.16E-04	6.59E-05	1.38E-04	1.33	ES	1.71E-02
Proteobacteria	Escherichia-Shigella	6.82E-03	1.45E-02	5.48E-03	1.14E-02	1.24	ES	2.09E-02

Proteobacteria	Haemophilus	6.81E-04	7.31E-04	2.77E-03	1.49E-02	0.25	N-ES	2.22E-02
Firmicutes	Streptococcus	7.78E-03	5.95E-03	7.15E-03	1.56E-02	1.09	ES	2.44E-02
Proteobacteria	Ellin6055	4.32E-04	3.91E-04	7.57E-04	6.56E-04	0.57	N-ES	2.58E-02
Firmicutes	Oscillibacter	9.17E-04	9.78E-04	6.49E-04	7.13E-04	1.41	ES	2.84E-02
Acidobacteria	uncultured_o_Subgroup_2	1.26E-04	1.00E-04	8.26E-05	7.34E-05	1.52	ES	3.09E-02
Firmicutes	[Ruminococcus]_torques_group	3.94E-03	4.79E-03	5.86E-03	6.19E-03	0.67	N-ES	3.29E-02
Firmicutes	Mitsuokella	5.29E-04	1.91E-03	4.26E-03	2.60E-02	0.12	N-ES	3.49E-02
Bacteroidetes	Odoribacter	5.62E-04	3.77E-04	5.20E-04	6.89E-04	1.08	ES	4.63E-02
Firmicutes	Coprococcus_2	2.84E-03	6.48E-03	4.67E-03	1.15E-02	0.61	N-ES	4.85E-02

Table S6. Differential fecal metabolites between ES and N-ES groups.

Metabolite	ES		N-ES		Fold change (ES/N-ES)	Enrichment	P value	VIP
	Mean	SD	Mean	SD				
Phenylacetylglutamine	4.58E-01	1.05E+00	8.09E-02	1.45E-01	5.66	ES	2.70E-02	1.25685
Neopterin	1.04E-01	2.55E-01	1.87E-02	1.66E-02	5.57	ES	3.80E-02	1.08822
Mesna	4.75E-01	1.04E+00	8.64E-02	5.40E-02	5.49	ES	2.14E-02	1.21883
Isocorydine	2.56E+00	6.15E+00	4.81E-01	8.05E-01	5.33	ES	3.71E-02	1.40986
Prolylhydroxyproline	1.82E-01	3.84E-01	3.41E-02	4.01E-02	5.32	ES	1.81E-02	1.32542
Codamine	6.68E-02	1.55E-01	1.29E-02	2.15E-02	5.17	ES	3.20E-02	1.34651
N-Methyl-a-aminoisobutyric acid	1.36E+01	2.52E+01	2.82E+00	4.67E+00	4.83	ES	9.37E-03	1.61443
8-Hydroxycarteolol	1.64E-01	2.82E-01	3.58E-02	3.72E-02	4.58	ES	5.51E-03	1.42802
p-Cresol sulfate	1.20E+01	2.78E+01	2.63E+00	5.31E+00	4.57	ES	3.93E-02	1.32294
Cinncassiol D4 2-glucoside	4.40E-02	5.10E-02	9.88E-03	1.46E-02	4.45	ES	1.14E-04	1.05536
Uric acid	4.78E-01	8.45E-01	1.07E-01	2.21E-01	4.45	ES	8.90E-03	1.7749
8-Prenylphaseollinisoflavan	4.36E-02	6.17E-02	1.01E-02	8.24E-03	4.32	ES	1.05E-03	1.40237
Succinic acid	4.38E+01	6.38E+01	1.02E+01	7.04E+00	4.29	ES	1.42E-03	1.11057
(+)-2,3-Dihydro-3-methyl-1H-pyrrole	1.45E+00	3.12E+00	3.41E-01	2.72E-01	4.26	ES	2.79E-02	1.25007
5-(2-Furanyl)-3,4-dihydro-2H-pyrrole	1.15E+00	2.31E+00	2.76E-01	2.25E-01	4.15	ES	2.03E-02	1.08183
Kanzonol V	1.64E-02	2.70E-02	3.97E-03	2.80E-03	4.14	ES	4.84E-03	1.32723
Aspartyl-Valine	3.39E-01	4.78E-01	8.29E-02	2.91E-01	4.09	ES	4.85E-03	1.8291
Leucyl-phenylalanine	1.22E-01	1.78E-01	2.99E-02	3.46E-02	4.08	ES	1.93E-03	1.36983
Kuwanol C	4.26E-03	4.14E-03	1.05E-03	1.08E-03	4.07	ES	9.11E-06	1.84031
5-Butyloxazole	9.64E-01	1.47E+00	2.38E-01	2.03E-01	4.05	ES	2.83E-03	1.01059
3-(2-Furanylmethylene)pyrrolidine	1.47E-01	3.20E-01	3.80E-02	4.57E-02	3.86	ES	3.67E-02	1.0275
2-Amino-3,8-dimethyl-3H-imidazo[4,5-f]quinoxaline	2.00E-01	2.78E-01	5.19E-02	5.04E-02	3.85	ES	1.43E-03	1.1431
Flazine	1.94E-01	4.07E-01	5.07E-02	4.95E-02	3.83	ES	2.97E-02	1.34784
beta-Alanine	4.06E-01	7.82E-01	1.08E-01	4.13E-02	3.76	ES	1.85E-02	1.67819
20,24-Epoxy-25,26-dihydroxydammaran-3-one	7.20E-02	1.20E-01	1.97E-02	1.84E-02	3.65	ES	8.00E-03	1.51952
D-Alanyl-D-alanine	5.82E-01	8.78E-01	1.61E-01	1.55E-01	3.62	ES	3.73E-03	1.40805
Cavipetin C	1.15E-01	2.12E-01	3.37E-02	2.16E-02	3.42	ES	1.79E-02	1.0099

2-Methoxy-3-methylpyrazine	1.13E+00	2.28E+00	3.33E-01	3.26E-01	3.40	ES	3.10E-02	1.14088
L-Glutamic acid	2.55E-01	5.16E-01	7.58E-02	6.66E-02	3.36	ES	3.29E-02	1.12337
20-Carboxy-leukotriene B4	2.86E-02	3.13E-02	8.62E-03	7.18E-03	3.32	ES	1.76E-04	1.39232
S-Acetyldihydrolipoamide	2.35E-01	2.45E-01	7.10E-02	3.87E-02	3.30	ES	7.66E-05	1.31269
L-Histidinol	2.36E+00	2.41E+00	7.36E-01	1.35E+00	3.21	ES	3.64E-04	1.66969
Ethyl acetoacetate	2.07E-01	2.50E-01	6.48E-02	3.64E-02	3.19	ES	6.35E-04	1.07951
Sucrose acetate isobutyrate	1.20E-02	1.27E-02	3.79E-03	3.81E-03	3.17	ES	1.87E-04	1.5798
Withaperuvin F	5.32E-02	7.84E-02	1.71E-02	3.69E-02	3.11	ES	1.02E-02	1.18087
Cer(d18:0/14:0)	1.64E-01	2.00E-01	5.28E-02	6.68E-02	3.10	ES	1.32E-03	1.16177
25,27-Dihydro-4,7-didehydro-7-deoxyphysalin A	5.32E-02	1.01E-01	1.73E-02	1.91E-02	3.08	ES	2.93E-02	1.42641
Calystegine B5	1.33E-01	2.07E-01	4.41E-02	4.65E-02	3.03	ES	9.42E-03	1.30194
1-Methyl-2-propylbenzene	2.76E-01	5.74E-01	9.17E-02	3.39E-02	3.01	ES	4.58E-02	1.19894
N-Acetylornithine	3.12E-01	6.50E-01	1.05E-01	7.31E-02	2.98	ES	4.82E-02	1.42705
Methyldopa	2.37E-01	4.71E-01	8.16E-02	8.61E-02	2.90	ES	4.35E-02	1.01077
(R)-3-Hydroxybutyric acid	4.28E-02	2.06E-02	1.49E-02	4.06E-03	2.88	ES	1.38E-12	1.68683
2-(4-Methylphenyl)propanal	5.95E-01	1.04E+00	2.13E-01	1.13E-01	2.80	ES	2.39E-02	1.17829
(-)-erythro-Anethole glycol 2-glucoside	1.57E-01	2.55E-01	5.68E-02	3.77E-02	2.77	ES	1.58E-02	1.23143
Fructosamine	4.35E-01	3.74E-01	1.58E-01	1.58E-01	2.76	ES	4.48E-05	1.34219
11(R)-HETE	2.74E+00	3.96E+00	9.95E-01	7.34E-01	2.75	ES	7.50E-03	1.01845
Asparaginyl-Proline	1.50E+00	2.82E+00	5.45E-01	3.59E-01	2.75	ES	3.76E-02	1.35698
Methyl 3-(methylthio)butanoate	4.97E-01	4.51E-01	1.81E-01	1.07E-01	2.74	ES	4.90E-05	1.37198
1-Phenyl-1,3-eicosanedione	6.79E-02	7.00E-02	2.50E-02	1.84E-02	2.72	ES	3.39E-04	1.45211
N-a-Acetyl-L-arginine	1.08E+00	1.78E+00	4.00E-01	3.75E-01	2.71	ES	1.99E-02	1.52015
3alpha,4,5,7alpha-Tetrahydro-5-hydroxy-1H-isoindole-1,3(2H)-dione	3.56E-01	5.75E-01	1.32E-01	1.75E-01	2.70	ES	2.12E-02	1.09733
Imidazoleacetic acid	1.25E+01	2.26E+01	4.64E+00	3.39E+00	2.69	ES	3.29E-02	1.03471
N-Hexadecanoylpyrrolidine	1.17E-01	1.78E-01	4.44E-02	1.02E-01	2.64	ES	2.73E-02	1.00605
Ecgonine	2.79E-01	5.25E-01	1.06E-01	1.32E-01	2.64	ES	4.59E-02	1.14975
Succinic semialdehyde	7.59E+00	6.01E+00	2.88E+00	1.46E+00	2.64	ES	6.93E-06	1.24287
N,N-Dimethylsphingosine	7.26E-01	1.07E+00	2.76E-01	6.44E-01	2.63	ES	2.51E-02	1.02085
Acetylhomoserine	1.79E-01	2.28E-01	6.86E-02	4.06E-02	2.61	ES	3.37E-03	1.22086
Alanyl-Proline	4.00E-01	5.76E-01	1.54E-01	1.08E-01	2.60	ES	9.61E-03	1.15366

Pipecolic acid	9.07E+00	7.53E+00	3.52E+00	1.67E+00	2.58	ES	1.92E-05	1.10496
Plastoquinone 3	3.48E-02	4.58E-02	1.36E-02	1.69E-02	2.57	ES	7.24E-03	1.54318
Anabsinthin	7.39E-02	9.45E-02	2.90E-02	1.16E-02	2.55	ES	3.81E-03	1.14821
5-Nitro-2-propoxyaniline	1.54E-01	2.27E-01	6.07E-02	4.58E-02	2.53	ES	1.29E-02	1.05835
1,2,3,4,5,6-Hexahydro-5-(1-hydroxyethylidene)-7H-cyclopenta[b]pyridin-7-one	1.17E+00	1.93E+00	4.61E-01	3.40E-01	2.53	ES	2.61E-02	1.0251
N-Acetylvanilalanine	1.61E-01	2.52E-01	6.42E-02	5.27E-02	2.51	ES	1.99E-02	1.01382
L-Malic acid	5.34E+00	3.58E+00	2.15E+00	2.14E+00	2.48	ES	6.63E-06	1.72831
Deoxyadenosine	1.27E+01	2.21E+01	5.15E+00	5.08E+00	2.47	ES	3.81E-02	1.0351
Dihydroisoalantolactone	2.96E-01	4.02E-01	1.21E-01	1.08E-01	2.43	ES	9.77E-03	1.0259
gamma-Aminobutyric acid	6.01E+00	9.74E+00	2.52E+00	3.69E+00	2.38	ES	3.76E-02	1.19247
Stigmast-22-ene-3,6-dione	4.12E+00	4.59E+00	1.82E+00	8.19E-01	2.27	ES	2.48E-03	1.07688
3-Butyl-1(3H)-isobenzofuranone	8.39E-01	1.13E+00	3.70E-01	1.98E-01	2.27	ES	1.18E-02	1.16221
L-Valine	9.86E+00	3.98E+00	4.36E+00	4.86E+00	2.26	ES	3.92E-07	1.73538
(2S,2'S)-Pyrosaccharopine	4.47E-01	7.58E-01	1.99E-01	1.40E-01	2.25	ES	4.52E-02	1.03992
PC(18:2(9Z,12Z)/18:0)	5.65E+00	6.53E+00	2.52E+00	2.53E+00	2.24	ES	6.06E-03	1.02837
Furanofukinin	1.90E-01	2.24E-01	8.54E-02	4.89E-02	2.23	ES	4.97E-03	1.12466
Deoxyinosine	3.51E-01	4.36E-01	1.58E-01	1.78E-01	2.22	ES	1.13E-02	1.05121
Kiwiionoside	1.03E-02	9.71E-03	4.64E-03	3.94E-03	2.22	ES	1.02E-03	1.59571
3-(4-Methoxyphenyl)-2-methyl-2-propenal	3.18E-01	4.21E-01	1.44E-01	8.55E-02	2.21	ES	1.22E-02	1.1184
N-(Heptan-4-yl)benzo[d][1,3]dioxole-5-carboxamide	1.27E-01	9.97E-02	5.76E-02	5.60E-02	2.21	ES	2.39E-04	1.34448
Histidinyl-Glutamine	1.80E-02	1.66E-02	8.17E-03	1.08E-02	2.21	ES	2.37E-03	1.5254
3-(2-Hydroxy-4-methylphenyl)-2-butanone	1.10E-01	1.41E-01	4.98E-02	1.76E-02	2.20	ES	9.33E-03	1.01522
DL-Glutamate	8.37E+00	4.81E+00	3.83E+00	2.11E+00	2.19	ES	5.29E-07	1.2281
L-Glutamine	1.71E-01	2.27E-01	7.84E-02	7.89E-02	2.19	ES	1.69E-02	1.68567
N-Acetylvaline	2.19E+00	3.02E+00	1.01E+00	1.40E+00	2.18	ES	2.69E-02	1.00162
Oxoglutaric acid	2.99E+00	2.64E+00	1.38E+00	7.04E-01	2.16	ES	3.77E-04	1.14374
L-Phenylalanine	1.71E+01	1.62E+01	8.06E+00	1.03E+01	2.12	ES	3.77E-03	1.65261
Pyruvic acid	4.29E+00	4.48E+00	2.14E+00	1.43E+00	2.00	ES	5.06E-03	1.02216
(3S,3'R,5R,6R)-7',8'-Didehydro-3,6-epoxy-5,6-dihydro-beta,beta-carotene-3',5-diol	1.49E+00	1.68E+00	7.45E-01	3.62E-01	1.99	ES	7.85E-03	1.14768
13-Tetradecene-1,3-diyne-6,7-diol	7.40E-01	7.64E-01	3.75E-01	2.64E-01	1.97	ES	5.51E-03	1.02669

5-O-Methylembelin	7.89E-02	1.07E-01	4.02E-02	3.12E-02	1.96	ES	3.10E-02	1.0098
3,4-Dihydro-6-hydroxy-2,5,7,8-tetramethyl-2H-1-benzopyran-2-carboxylic acid	1.67E-01	1.94E-01	8.72E-02	8.00E-02	1.92	ES	1.80E-02	1.0813
Santalyl phenylacetate	3.94E-02	3.34E-02	2.12E-02	1.32E-02	1.86	ES	1.94E-03	1.08342
Spirostane-3,6-dione	3.34E-02	3.49E-02	1.84E-02	1.50E-02	1.82	ES	1.46E-02	1.09464
Lutein	4.54E+00	2.65E+00	2.51E+00	1.29E+00	1.81	ES	3.73E-05	1.16406
L-Lysine	4.61E+00	4.94E+00	2.62E+00	2.66E+00	1.76	ES	2.77E-02	1.20809
23-Acetoxysoladulcidine	7.21E-02	4.86E-02	4.36E-02	3.73E-02	1.65	ES	4.33E-03	1.33263
5-Hydroxybuspirone	1.49E-02	1.04E-02	9.02E-03	8.39E-03	1.65	ES	6.93E-03	1.14604
1-Methyladenosine	3.19E-01	2.79E-01	1.96E-01	2.33E-01	1.62	ES	3.64E-02	1.0381
8-iso-15-keto-PGE2	9.21E-02	1.52E-01	1.48E-01	4.57E-02	0.62	N-ES	3.04E-02	1.18669
Armillane	4.70E-03	6.21E-03	7.92E-03	2.27E-03	0.59	N-ES	2.83E-03	1.12072
alpha-Acetolactate decarboxylase (enzyme preparation from bacillus subtilis recombinant)	8.39E-03	9.76E-03	1.44E-02	4.49E-03	0.58	N-ES	7.36E-04	1.26167
Threoninyl-Arginine	7.41E-02	6.41E-02	1.34E-01	3.80E-02	0.55	N-ES	2.70E-06	1.34536
Santene	3.81E-01	2.97E-01	6.92E-01	5.60E-02	0.55	N-ES	6.68E-09	1.02351
Anhydrocinnzeylanine	5.56E-03	4.34E-03	1.03E-02	3.49E-03	0.54	N-ES	8.75E-07	1.06038
1-Isopropyl-2,3-dimethylcyclopentane	2.88E-02	3.16E-02	5.34E-02	2.03E-02	0.54	N-ES	8.67E-05	1.318
L-Serine	6.31E-01	4.53E-01	1.19E+00	4.44E-01	0.53	N-ES	2.84E-07	1.04199
6-Epi-7-isocucurbic acid glucoside	3.07E-02	2.50E-02	5.82E-02	1.64E-02	0.53	N-ES	1.24E-07	1.06333
4-Hydroxy-5-phenyltetrahydro-1,3-oxazin-2-one	4.57E-02	3.41E-02	8.73E-02	2.16E-02	0.52	N-ES	6.35E-09	1.23989
Glutamylvaline	4.96E-02	6.74E-02	9.64E-02	2.15E-02	0.51	N-ES	7.50E-05	1.19408
L-Theanine	1.96E-01	1.99E-01	3.96E-01	1.28E-01	0.49	N-ES	7.71E-07	1.25968
Cincassiol B	8.12E-03	4.23E-03	1.68E-02	4.14E-03	0.48	N-ES	2.90E-14	1.07696
10'-Apo-beta-caroten-10'-al	2.74E-01	2.28E-01	5.82E-01	1.64E-01	0.47	N-ES	1.05E-09	1.25675
Heteroflavanone C	6.16E-03	7.08E-03	1.31E-02	4.44E-03	0.47	N-ES	1.11E-06	1.44589
Isoleucyl-Aspartate	3.95E-01	2.67E-01	8.56E-01	2.22E-01	0.46	N-ES	1.55E-12	1.20301
Glycine	1.04E-02	1.75E-02	2.28E-02	2.66E-02	0.46	N-ES	1.66E-02	1.13155
8-Hydroxyamoxapine	4.41E-03	6.27E-03	9.75E-03	9.30E-03	0.45	N-ES	3.52E-03	1.03966
Kanzonol M	6.08E-03	5.04E-03	1.40E-02	3.71E-03	0.43	N-ES	1.02E-11	1.22333

Isocitric acid	3.09E-01	4.08E-01	7.49E-01	8.11E-01	0.41	N-ES	3.02E-03	1.32515
Vanillylmandelic acid	9.06E+00	1.10E+01	2.32E+01	1.42E+01	0.39	N-ES	3.65E-06	1.72461
Citrulline	1.93E+00	1.17E+00	5.04E+00	1.50E+00	0.38	N-ES	2.98E-16	1.36519
Gentisate aldehyde	1.76E+00	1.18E+00	4.98E+00	2.09E+00	0.35	N-ES	1.03E-12	1.68999
Taurine	7.28E-02	1.39E-01	2.11E-01	1.83E-01	0.34	N-ES	2.71E-04	2.72175
Floribundoside	5.45E-03	1.27E-02	1.95E-02	2.80E-02	0.28	N-ES	4.89E-03	1.73882
Naringin	2.07E-02	4.62E-02	8.48E-02	1.24E-01	0.24	N-ES	2.94E-03	1.45171
D-Xylose	7.37E-02	1.69E-01	3.06E-01	5.43E-01	0.24	N-ES	1.16E-02	1.0285
Hesperidin	4.55E-01	9.28E-01	1.98E+00	2.66E+00	0.23	N-ES	1.02E-03	1.08222
Naringenin	4.65E-02	1.04E-01	2.02E-01	2.81E-01	0.23	N-ES	1.47E-03	1.71369
Chalconosakuranetin	2.85E-01	5.99E-01	1.25E+00	1.71E+00	0.23	N-ES	1.22E-03	1.10061
Ketoprofen glucuronide	1.21E-01	2.61E-01	5.43E-01	7.46E-01	0.22	N-ES	1.16E-03	1.2497
Hesperetin 7-glucoside	1.09E-01	2.29E-01	4.99E-01	6.92E-01	0.22	N-ES	1.13E-03	1.46578
3,6,7-Trihydroxy-4'-methoxyflavone 7-rhamnoside	1.76E-02	3.81E-02	8.14E-02	1.13E-01	0.22	N-ES	1.12E-03	1.48788
3,4-Dimethoxybenzoic acid	9.33E-01	1.68E+00	4.40E+00	8.46E+00	0.21	N-ES	1.31E-02	1.39883
Hesperetin	5.45E-01	1.19E+00	2.64E+00	3.87E+00	0.21	N-ES	1.58E-03	1.53195
Deoxycytidine	9.75E-02	1.99E-01	5.03E-01	8.31E-01	0.19	N-ES	3.58E-03	1.2294
